# Novel method for rubber hand illusion strength measurement based on inverse multidimensional scaling

**DOI:** 10.3758/s13428-025-02900-2

**Published:** 2025-12-02

**Authors:** Piotr Litwin, Katarzyna Kubik, Matthew R. Longo

**Affiliations:** 1 Faculty of Psychology, University of Warsaw, Banacha 2D, 02-097 Warsaw, Poland; 2 Faculty of Philosophy, University of Warsaw, Krakowskie Przedmieście 3, 00-927, Warsaw, Poland; 3 School of Psychological Sciences, Birkbeck, University of London, Malet St, London, WC1E 7HX UK

**Keywords:** Rubber hand illusion, Inverse multidimensional scaling, Body ownership, Illusion strength measurement

## Abstract

In the present study, we developed a novel self-report measurement method for the rubber hand illusion (RHI) strength based on inverse multidimensional scaling (MDS). In the preregistered study consisting of two experiments, participants experienced the RHI in synchronous and asynchronous conditions (Experiment 1) as well as the RHI and the arm immobilization imaginative suggestion (Experiment 2). In each condition, participants repeatedly arranged items related to distinct bodily-related experiences (including RHI or suggestion) in accordance with the perceived similarity between them. Proximity data obtained from the arrangements were represented as distances in the multidimensional bodily space. To measure RHI strength, we focused on distances between items representing experimental conditions and two baseline items representing cases of no ownership over an external object and normal bodily feelings. We found that the distance between the rubber hand and an external object was significantly larger in the synchronous than the asynchronous condition, and larger than the distance between the immobilized arm and the normal body, demonstrating stronger shifts in ownership for synchronous RHI. In general, the RHI was associated with moderate ownership and low perceived stimulation, and it clustered with experiences related to a high degree of ownership. MDS-based solutions for the bodily space were consistent within participants and across different experimental conditions. We believe that this method can complement traditional questionnaire-based measurement, offering additional opportunities for a comprehensive self-assessment of RHI strength.

**Supplementary Information:**

The online version contains supplementary material available at 10.3758/s13428-025-02900-2.

In the rubber hand illusion (RHI; Botvinick & Cohen, 1998), participants observe a rubber hand being stroked synchronously and in a spatially congruent manner with their own visually occluded hand. This procedure induces a convincing impression that tactile sensations arise from the rubber hand, as if it were a part of one’s body. As evidenced by spontaneous remarks and self-reports in early research (Armel & Ramachandran, 2003; Botvinick & Cohen, 1998), as well as by qualitative studies (Bartoletti et al., 2023; Lewis & Lloyd, 2010; Valenzuela Moguillansky et al., 2013), participants report that the vividness of the illusion varies across experimental conditions and may fluctuate over time, depending on the properties of multisensory stimulation. These multiple reports led to the intuition that the RHI, like most perceptual illusions, has a quantifiable “strength” which can be defined as the magnitude of a non-veridical perceptual skew from the real parameter value. In this case, we can define RHI strength as the magnitude of a non-veridical gain in feelings of bodily ownership over an external object (i.e., rubber hand), while in reality there is no actual ownership over this object.

Unlike some other illusions, the exact strength of the RHI is problematic to measure. For example, in many visual illusions, the real parameter (e.g., length) value is observable and can be measured using appropriate instruments (e.g., a measuring tape), while perceptual bias can be reported by the participants in a relatively controlled response manner (e.g., through reproduction or a graded series scale) (Todorović, 2020). This opens up the possibility of calculating the illusion strength index as the difference between what was perceived and what was presented. In contrast, in the RHI, ownership feelings cannot be presented to a participant as a stimulus and are only indirectly related to measurable properties of visuo-tactile stimulation. Thus, these latent ownership feelings cannot be approximated through reproduction or matching to a reference ownership value.

Researchers investigating changes in RHI strength most often rely on seven-point Likert scales. Participants are typically asked to indicate their agreement with statements about possible experiences on a scale ranging from − 3 (strongly disagree) to 3 (strongly agree). A brief questionnaire consisting of nine such statements—three addressing the experience during the illusion and six control statements not directly related to actual experiences of the RHI—was introduced in the original paper on the illusion (Botvinick & Cohen, 1998) and continues to be used in its original form (Tosi et al., 2024). Such rating scales and short questionnaires have several important advantages: they are easy to administer, provide a straightforward affirmation-versus-denial interpretation (with positive scores indicating agreement, negative scores indicating disagreement, and intermediate values capturing uncertainty), and have enabled a rapid accumulation of knowledge about the multisensory determinants of the RHI and related bodily illusions (Ehrsson, 2020; Litwin, 2020), as well as their expression in clinical populations (Baum et al., 2022; Brizzi et al., 2023).

When applied in extended form, questionnaires also enable a quantitative assessment of the multifaceted phenomenological structure of the RHI. Using a psychometric approach, Longo et al. (2008) examined 27 statements derived from qualitative descriptions of subjective experiences during the illusion. Principal component analysis identified four components: *embodiment of the rubber hand, loss of one’s own hand*, *movement*, and *affect*. While the *movement* factor largely comprised statements routinely used as control items, and *affect* reflected the valence of the experience, the first two components corresponded directly to illusion strength. A secondary analysis of the main embodiment component revealed three aspects: feelings of the rubber hand being part of one’s body (*ownership*), the overlap of proprioceptive and tactile sensations from both hands (*location*), and the sense of being able to control the rubber hand (*agency*). The same 27 statements were later re-examined by Romano et al. (2021), who allowed the components to be correlated and obtained a simpler solution consisting of three major components: *embodiment of the rubber hand*, *disembodiment*, and *physical sensations*. Notably, the first two components largely overlapped with Longo et al.’s (2008) solution and were significantly correlated. Items that significantly loaded on one of the three components were then averaged to form an embodiment scale with satisfactory psychometric properties; within this scale, the *embodiment* subscale proved most sensitive to differences between experimental and control conditions. Finally, a study employing exploratory graph analysis showed that ownership experiences can be distinguished from referral-of-touch experiences, which—although correlated with ownership—relate specifically to the perceived origin of tactile sensations (Tosi et al., 2024). These findings corroborated earlier reports that participants more readily affirm referral-of-touch responses (Kalckert et al., 2019; Reader et al., 2021a), potentially suggesting that the three original statements introduced by Botvinick and Cohen (1998) may capture different aspects of the RHI.

While psychometric approaches to the RHI have provided valuable insights into the subtleties of ownership-related experiences, research practice shows that validated questionnaires are rarely the method of choice. Instead, researchers predominantly rely on shorter rating scales, with individual studies differing in fundamental aspects of questionnaire-based measurement, such as the selection of experimental and control items or the specific method of operationalizing RHI strength (Riemer et al., 2019). Some have analyzed different experiential dimensions (e.g., referral of touch, ownership, and disownership) separately, either as independent indices (e.g., Kalckert et al., 2019) or as individual statements (e.g., Reader et al., 2021b). Others have calculated a general index, either by pooling several experimental statements (e.g., Radziun & Ehrsson, 2018) or by subtracting pooled experimental statements from averaged control statements (e.g., Burin et al., 2019). Still others have introduced an “illusion present” cutoff point based on the value of an averaged index (e.g., ≥ 1 on a seven-point scale ranging from − 3 to 3; Kalckert & Ehrsson, 2012). These methodological choices may contribute to substantial variability in the reported results (Riemer et al., 2019) and, potentially, in the conclusions drawn.

One reasonable approach to addressing these issues would be to establish a standard questionnaire-based measure in the field—for example, by constructing an RHI strength scale with reliable psychometric properties (cf. Romano et al., 2021) and a clearly defined scoring system. However, such an approach may overlook the richer phenomenology of the illusion. Previous efforts have yielded psychometric solutions that account for only about 50% of the variance (Longo et al., 2008: 55.3%; Romano et al., 2021: 48%), with the embodiment component explaining roughly 25% (Longo et al., 2008: 26.3%; Romano et al., 2021: 24%). From a broader measurement perspective, RHI strength assessed with rating scales may also be vulnerable to error stemming from unmet assumptions of Likert-scale use. Psychometric studies indicate that the classic assumption of equidistant, interval thresholds in Likert ratings often fails to hold (Hilbert et al., 2016; Knutsson et al., 2010; Sideridis et al., 2023), unless the reported latent state is familiar, frequently experienced, and graded—for example, in the case of positive affect (cf. Sözer & Kahraman, 2021). In personality scales, successive pairs of Likert steps are separated by unequal distances, and this property varies greatly across items (García-Pérez, 2024). This pattern is consistent with item response theory (Embretson & Reise, 2013), which conceptualizes the mapping from continuous latent variables to observed responses as nonlinear and governed by item-specific thresholds, which may manifest differently across individuals depending on their latent trait level.

These methodological considerations have important implications for RHI measurement. In RHI studies, participants often report intermediate ownership experiences (as evidenced by mean self-report scores that rarely approach the maximum; Reader et al., 2021a), whereas in everyday life, ownership is typically stable and not experienced as disturbed or unusual. This discrepancy raises the risk that the mapping between latent ownership and scale response thresholds is nonlinear and uncertain, particularly in the intermediate range. Variable decision thresholds may introduce bias, resulting in systematic error that depends on group assignment or condition, thereby increasing the likelihood of type I or type II errors and threatening the construct validity of the scale (King & Wand, 2007). In the domain of perceptual illusions, this fact was evidenced by the study employing the Müller–Lyer illusion (Coren & Girgus, 1972). In that study, participants used several methods to assess the perceived relative length of two shafts, one of which was a seven-point Likert scale ranging from “very much larger” to “very much smaller.” Unlike other methods, the rating scale failed to detect significant differences between conditions with varying wing angles, despite the fact that these differences are easily visually perceivable. Thus, the rating scale misrepresented the perceptual reality of the illusion, likely due to the unclear mapping between the continuous variable of perceived relative length and the discrete, and potentially ambiguous, categories of the scale (e.g., “much larger”). In the RHI paradigm, analogous concerns are most often alleviated by the use of within-subject designs and the calculation of indices based on condition-specific differences (e.g., synchronous vs. asynchronous stimulation). These indices correlate with other measures of the illusion, such as proprioceptive drift (Tosi et al., 2023), but not with external factors mainly influencing scale responding, such as suggestibility (Ehrsson et al., 2022), which supports the construct validity of this mode of questionnaire-based measurement.

While Likert scales remain a valid and widely used method for differential RHI measurements, the body ownership field could still benefit from developing a complementary self-assessment tool providing additional self-report options alongside questionnaires. The motivation of the present studies was to make the mapping between the continuous latent variable (i.e., feelings of ownership) and the response space clearer by using intersubjectively understandable items related to common experiences of fluctuations in bodily feelings, including changes in ownership. In this way, participants were able to meaningfully compare the RHI to familiar bodily experiences differing in terms of touch, bodily control, and general sensations. We reasoned that such an alternative response format could both stabilize the response space and capture aspects of ownership experience that standard scales assess less effectively. By broadening the methodological toolkit available to researchers, an additional self-report approach could accelerate future research on bodily illusions by enabling robust cross-validation with both questionnaire-based and indirect (i.e., behavioral or physiological) measures of RHI strength.

Instead of using scales, we employed the inverse multidimensional scaling (MDS) method (Kriegeskorte & Mur, 2012) to obtain individual proximity data on multiple body-related experiences (including the RHI) and represent them as distances in a multidimensional bodily space. Adopting this bottom-up approach to constructing psychological bodily space allowed us to experimentally disentangle its dimensions in a less theory-driven manner, without the need to constrain the spatial organization a priori or to impose a scale-like form. In the MDS framework, Euclidean distances between points in bodily space can be calculated regardless of the underlying data structure, providing an index of dissimilarity between experiences (e.g., between the RHI and ordinary full-body ownership).

The present study was inherently exploratory, as we aimed to provide a proof-of-concept for a new method of ownership measurement. Our exploration had several aims: First, we wanted to determine whether inverse MDS could serve as a useful method for measuring RHI strength. Second, we sought to identify which of two possible MDS-based measurement approaches would be more promising: (1) using normalized “ownership scores” based on point coordinates along bodily space dimensions interpretable in terms of ownership, or (2) calculating distances between RHI and two baseline items representing canonical cases—no ownership of an external object (the empty glove item) and full ownership (the normal bodily feelings item). Finally, we wanted to describe the structure of the bodily space in a bottom-up way, possibly to gain new insights regarding the phenomenology of body ownership and the RHI directly from participants’ similarity judgments. We were particularly interested in whether we could discern dimensions in and extract clusters from this multidimensional bodily space. However, despite its exploratory nature, we preregistered the study (https://osf.io/amzk4) for two reasons. First, we expected the proposed method would be sufficiently sensitive to differentiate RHI from control conditions. Therefore, we formulated four hypotheses based on distances between RHI and the baseline items representing experiences of no ownership and full ownership, which we could calculate for all participants regardless of their individual bodily space solutions. Second, we preregistered a detailed design, sampling, and analysis plan to constrain analytical possibilities and ensure consistency with best practices in the MDS field, aiming for robust and replicable results.

The study consisted of two experiments in a simple two-level within-subject design. In Experiment 1, participants experienced the RHI in the two most common conditions—synchronous and asynchronous visuo-tactile stimulation. We hypothesized that the rubber hand item would be closer to the normal bodily feelings item and farther from the empty glove item in the synchronous condition compared to the asynchronous condition. In Experiment 2, we replaced the asynchronous condition with the arm immobilization imaginative suggestion from the Sussex–Waterloo Scale of Hypnotizability (SWASH) battery (Lush et al., 2018). Arm immobilization experience includes a hypnotic suggestion that one’s arm becomes unable to move. We reasoned that for the MDS-based method to be useful for the purpose of ownership assessment, it should be less sensitive to (imaginary) ownership shifts stemming from suggestion than to (perceptual) ownership shifts stemming from multisensory stimulation. Because the suggestion works in the opposite direction to the RHI (i.e., during the suggestion, the participant is expected to temporarily “lose” command over one’s own hand), we hypothesized (1) that the rubber hand item would be closer in the bodily space to the normal bodily feelings item than the arm immobilization item to the empty glove item, and (2) that the rubber hand item would be farther from the empty glove item than the arm immobilization item was from the normal bodily feelings item.

## Method

### Participants

To determine the sample size appropriate for our study, we focused on the number of participants required for paired *t*-tests comparing distances of interest in particular conditions. For Experiment 1, we derived the expected effect size for a difference between synchronous and asynchronous conditions (*d* = 0.95) as well as the correlation between RHI strength in both conditions (*r* = 0.44) from one of the largest studies on the RHI (Lush et al., 2020) (data available at https://osf.io/q39kw). Required sample size was calculated for a one-sided paired-samples *t*-test assuming 0.9 power, which yielded *N* = 12. We decided to substantially increase this number to at least 25 participants, which we deemed sufficient to yield robust and generalizable bodily space solutions. The sample size for Experiment 2 was determined after Experiment 1 was conducted to also include no less than 25 participants.

Participants were students of various majors, recruited via the University of Warsaw’s anonymous platform. They were naïve to the study’s purpose and received course credit through a general credit system based on study participation, not through psychology classes. The experiment complied with the ethical guidelines specified in the Declaration of Helsinki, and was approved by the ethics committee of the Faculty of Psychology of the University of Warsaw.

### Stimuli

For participants to perform the inverse MDS task, we needed a set of widely familiar instances of transient changes in sensory perception of the body. Stimuli consisted of 13 colored images representing such bodily experiences, which from this point forward will be referred to as *items*. The first item depicted a hand model, interchangeably representing a particular experimental condition—either rubber hand (Exp. 1: both synchronous and asynchronous conditions; Exp. 2: synchronous condition) or one’s own hand (Exp. 2: arm immobilization suggestion condition, see *Tasks* below). Two other items represented two canonical cases of full ownership (“normal body feelings”) and no ownership feelings (“empty glove”). Ten further items represented uncommon ownership experiences, including cases of changed, partial, or disturbed ownership. As shown by studies using qualitative approaches (Bartoletti et al., 2023; Lewis & Lloyd, 2010; Valenzuela Moguillansky et al., 2013; also Longo et al., 2008, in a broader psychometric context), for some participants, the experience of the RHI, in either the synchronous or asynchronous condition, may be reminiscent of everyday examples of changed ownership feelings. On the basis of these studies, we selected 10 uncommon bodily experience items inspired by spontaneous participant remarks during qualitative interviews (for direct quotations and the studies from which particular items were derived, see Supplementary Material S1). Choice criteria for those uncommon bodily experience items required the experiences to be (1) related to changes in general bodily sensations, feelings, tactile experiences, or possibility to control, (2) possibly known for as many participants as possible, and (3) related more clearly to fluctuations in sense of ownership or sense of agency rather than to other forms of bodily experience (e.g., social signals, widespread emotional responses, or chronic pain). All items constituting the bodily space in the current study are presented in Table 1.

**Table 1:**
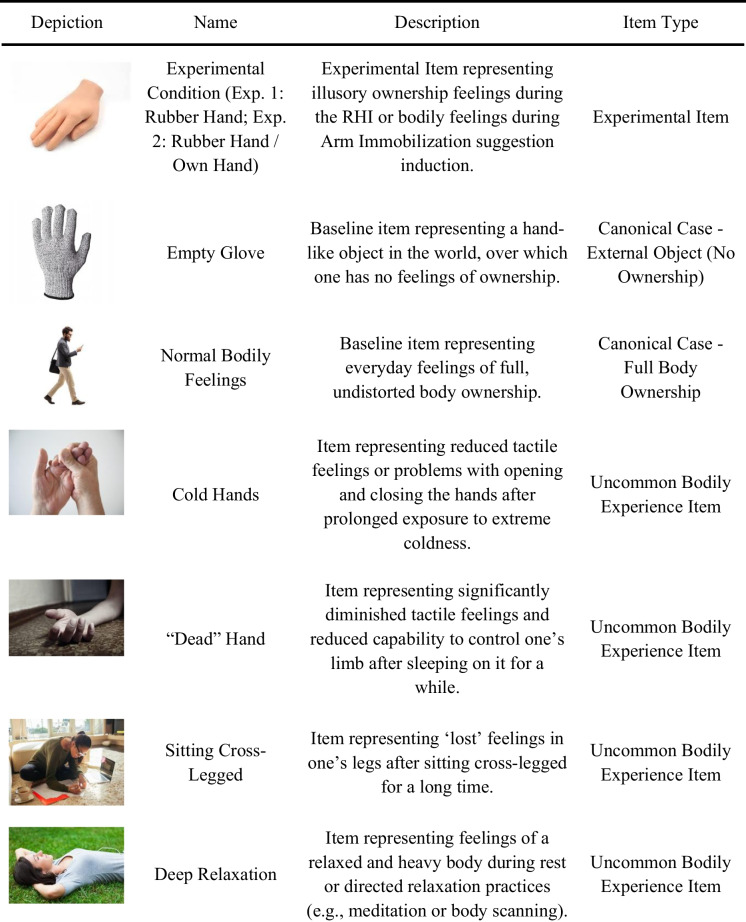

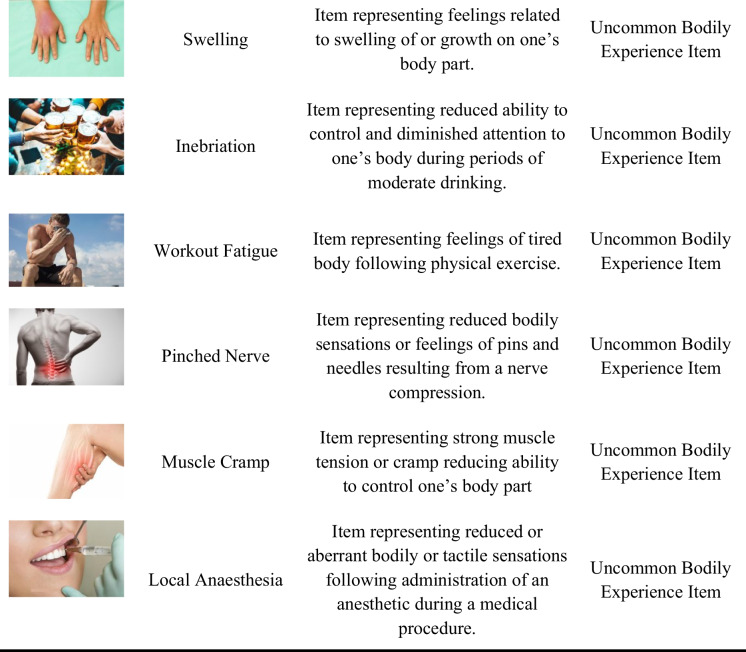
Items used in inverse MDS arrangements

### Tasks

#### Inverse multidimensional scaling

**Fig. 1:**
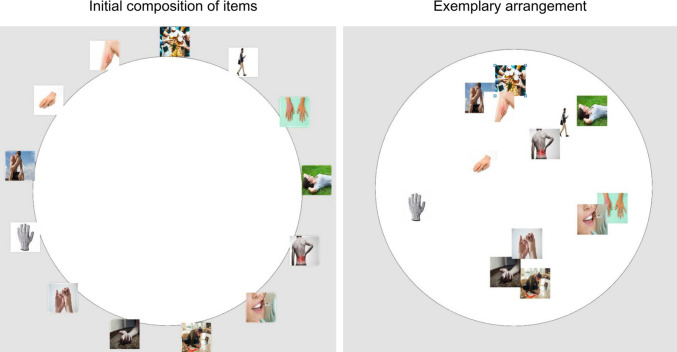
Inverse multidimensional scaling (MDS) method. *Note.* In inverse MDS, participants are presented with multiple items—constituting a mental space of interest—scattered in an initial composition (left panel). Participants’ task is to repeatedly arrange items in a circular space using mouse drag-and-drop, in such a way that similar items are bundled together and dissimilar items are separated. Pairwise distances translate directly to perceived (dis)similarity. The final arrangement (example; right panel) has to be confirmed with the use of a button press

Multidimensional scaling is a nonlinear method of dimensionality reduction that maps pairwise proximity (i.e., dissimilarity or distance) judgments into distances in a multidimensional space (Borg et al., 2018). The matrix of pairwise dissimilarities can then be visualized as a space in which distances between particular items reflect subjective dissimilarities between them. To circumvent the need to use overt dissimilarity ratings or abstract distance judgments, we used the inverse MDS method (Kriegeskorte & Mur, 2012) which works in the opposite direction: it infers a dissimilarity matrix from multiple item arrangements. Participants repeatedly arrange items in two-dimensional (2D) space by means of mouse drag-and-drop (Fig. 1). There is only a single rule organizing the arrangements: similar objects should be placed close together and dissimilar objects should be placed far apart. Multiple arrangements are performed using either the entire set or subsets of items, so as to allow the participant to possibly convey high-dimensional dissimilarity structure. An adaptive algorithm determines the number of items to be presented in consecutive trials, showing the entire set in the first trial, and optimally selecting subsets in further trials to maximize evidence for the least determined dissimilarity estimates. Finally, the evidence from multiple arrangements is statistically combined to obtain the representational dissimilarity matrix. These dissimilarity matrices can again be used to reconstruct individual or general (i.e., averaged across participants) bodily spaces using the standard MDS method.

As we aimed to obtain similarity judgments reflecting the natural representation of bodily space, we did not prompt participants to rely on any particular theoretical dimensions. Thus, no specific instructions on how to perform the arrangements were provided. The concept of body ownership was deliberately not introduced to participants, and canonical cases were not suggested as baseline values or reference points. To prevent task misunderstanding, participants were only instructed that we were exclusively interested in their feelings—that is, how they experience their body (or the rubber hand) in terms of general bodily sensations. The instructions also stressed that participants should not consider incidental item similarities not directly related to feelings and sensations (e.g., that both cold hands and dead hand items refer to hands) in their arrangements. Participants were also presented with three exemplary random arrangements, with arrows and circles illustrating how all distances between items directly translate to dissimilarities between them. Finally, the instructions stressed that all relative distances between all pairs of items matter and should therefore be double-checked before final approval. Instructions were standardized and presented in the form of a text displayed on the monitor (for exact instructions see Supplementary Material S2).

The inverse MDS procedure was run in MATLAB 2024A (MathWorks, Natick, MA, USA) using code developed by the authors of the method (Kriegeskorte & Mur, 2012). Parameters of the procedure were specified in accordance with the most recent contributions in the field (Jóźwik et al., 2022). An evidence utility exponent (E = 10) was applied to each of the stimuli to calculate stimulus utility in the event it was picked for the next trial. The procedure was terminated if the pair with the lowest evidence had an evidence weight higher than 0.5 or after participants completed 12 arrangements, whichever came first.

#### Rubber hand illusion

**Fig. 2:**
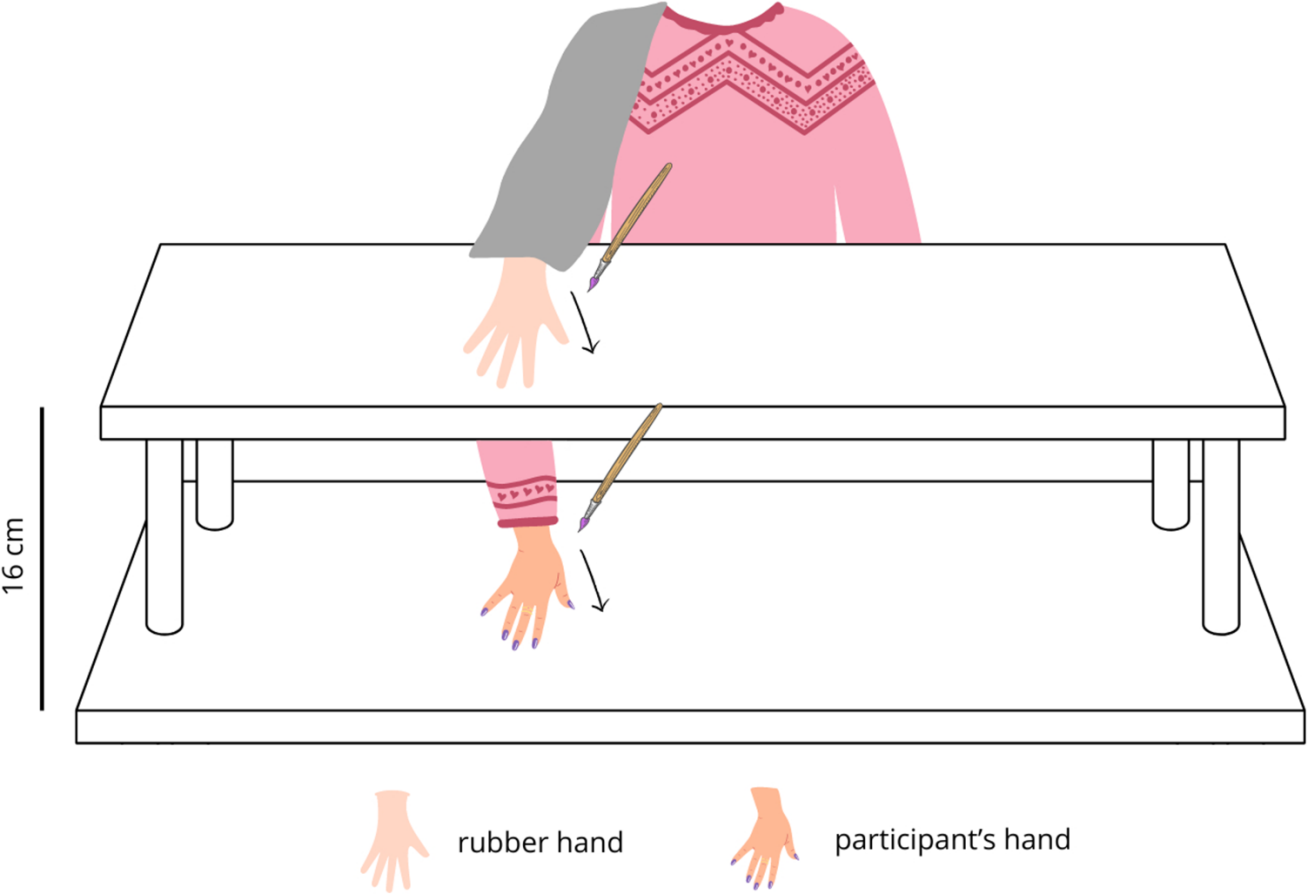
Schematic depiction of the experimental setup. *Note.* The rubber hand was placed on the upper level of the shelf, 16 cm in vertical axis from the participant’s hand placed on the lower level

For RHI elicitation, a very realistic, life-sized rubber right hand was used. The rubber hand was placed on the upper surface of a two-level shelf, directly in front of the participant’s right shoulder, and was clearly visible to the participant (Fig. 2). The space between the participant’s neck and the rubber hand was covered by a textile material. The participant’s hand was placed directly below on the lower level. Both hands were aligned horizontally and were separated by 16 cm in the vertical axis. The stimulation in both synchronous and asynchronous conditions lasted 90 s. In the synchronous condition, the RHI was elicited using a complex-structured visuo-tactile stimulation which tends to produce the most compelling illusion (Litwin et al., 2020). The index, middle, and ring fingers were stimulated through brushstrokes of varying length—extending from the proximal to the distal phalanges—and in an irregular pattern, with around 0.5 Hz frequency. In the asynchronous condition, we used an analogous spatiotemporal pattern, except that strokes were around 0.5 s out of phase.

#### Arm immobilization suggestion

For Exp. 2, we chose an imaginative suggestion (arm immobilization) from the Sussex–Waterloo Scale of Hypnotizability (SWASH; Lush et al., 2018) as a hand-related quasi-illusory counterpart experience to be induced in the control condition. Arm immobilization suggestion was selected as most closely resembling the disownership of one’s own hand, which phenomenological studies describe as a significant part of the RHI experience (Bartoletti et al., 2023; Lewis & Lloyd, 2010), and which psychometric studies suggest is the second main component of the RHI experience (Longo et al., 2008; Romano et al., 2021). Although the SWASH protocol requires prior induction of a para-hypnotic relaxation state to make participants more prone to the suggestions, we decided to remove this hypnotic relaxation to minimize contingent differences between the conditions. The RHI does not require participants to be in any specific state, and arguing that hypnotic relaxation is necessary for imaginative suggestion effects to occur would establish qualitative differences between the RHI and arm-related suggestions a priori. Arm immobilization suggestion was induced in accordance with the SWASH induction script and response booklet (available at https://osf.io/g72ae) after translation to Polish. Suggestion was paced in such a way that it took around 90 s to match induction times of RHI-related conditions. 

### Procedure

The study consisted of two experiments, each in a simple two-level within-subject design. In Experiment 1, participants experienced the RHI in the two most common conditions—synchronous and asynchronous. In Experiment 2, the asynchronous condition was replaced with the arm immobilization suggestion. The order of conditions was counterbalanced across participants. Immediately after having experienced either RHI or the suggestion, participants turned from the RHI setup table to the computer, which was on another desk, and completed a short questionnaire consisting of three ownership-related questions (cf. Litwin et al., 2020) derived from the Longo et al. (2008) study (Supplementary Table S3). In the imaginative suggestion condition, the questions were phrased as negative sentences (e.g. “(…) I felt as if my hand did not belong to me”) for higher ratings to stand for the stronger illusory experience, and to make the questions sound more natural to the participants. The questionnaire data on RHI strength were collected for the purpose of cross-validation and comparison with the MDS-based estimates.

After completing this short questionnaire, participants performed the inverse MDS task related to a particular condition. During the arrangements, participants were presented with a crib sheet including short descriptions of particular items to help them avoid possible confusion (full crib sheet, both in the original Polish version and translated to English, is included in Supplementary Material S4). The study part related to asynchronous RHI or arm immobilization conditions concluded upon completion of the inverse MDS task. In the synchronous RHI condition, participants were additionally presented with a short questionnaire related to their degree of familiarity with 10 uncommon bodily experience items (in Experiment 1, familiarity ratings from three participants were not collected due to software failure). Familiarity ratings were given on a five-point scale (see Supplementary Material S5 for the description of scale items). Both canonical cases and experiences represented by the experimental item were assumed to be completely familiar to the participants (i.e., their familiarity was set to the maximum value of 5). In each experiment, the whole procedure including both conditions took 30–40 min to complete.

### Data analysis

#### MDS solution assessment

MDS solutions were calculated using the *smacof* (Mair et al., 2022) and *vegan* (Oksanen et al., 2022) packages for R. Averaged dissimilarity matrices for general MDS solutions were calculated as means from individual dissimilarity matrices obtained in particular conditions. Interval MDS solutions were used under the assumption that they should lead to a more accurate structural representation of the interval distance data than ordinal solutions (Mair et al., 2016). The misfits of particular MDS configurations to the data were evaluated using the normalized Kruskal stress-1 value (Kruskal, 1964). Stress values were computed both for individual arrangements and for general solutions.

MDS solutions were selected and evaluated in terms of their goodness of fit in a principled, preplanned manner, following consecutive steps recommended in the literature (Mair et al., 2016) (cf. preregistration document at https://osf.io/amzk4). To find both individual and general (i.e., averaged) solutions with the lowest possible stress, we fitted 1,000 interval MDS solutions with multiple random starts in each analysis. Then, observed stress values were benchmarked against 500 random permutations of proximities within respective dissimilarity matrices. Solutions were deemed unsatisfactory and rejected if their stress value was not significantly lower, in a one-sided permutation test (*α* = 0.05), than the mean stress of MDS solutions fit to randomly permuted dissimilarities (i.e., if the observed stress was not lower than the fifth percentile in the distribution of stress values for permuted matrices). All solutions which passed the permutation test were additionally examined with respect to the rule of thumb for acceptable MDS fits (stress < 0.2; Hair et al., 2006). Solutions with stress higher than 0.2 were treated individually based on the exact stress value and the presence of peculiarities signaling potential task misunderstanding. In principle, we aimed to include data from as many participants as possible.

To assess the degree to which particular items contributed to the misfit of general MDS configurations, we computed stress-per-point (SPP) values for all items constituting the bodily space. In the case of detecting very high SPP values consistently for the same items, we planned to repeat the MDS fitting procedure described above with high SPP items eliminated from the bodily space.

Finally, we aimed to analyze the structure of the bodily space by interpreting dimensions of MDS solutions. Dimensions of all MDS solutions were subjected to non-principal axis rotations (Mair et al., 2015). Solutions were rotated in a way that minimized the values of normalized point coordinates associated with the baseline empty glove item on all dimensions in the space, in order to facilitate meaningful interpretation of these dimensions in terms of aspects of bodily experience. We also explored, using the *k*-means method, whether clusters related to different types of bodily experiences could be extracted from the MDS configurations. The optimal number of clusters for particular solutions was determined with the use of the *NbClust* R package (Charrad et al., 2014) as the number most often indicated among all calculated indices (i.e., through the “majority vote” rule). Derived dimensions and regions were assessed for stability across conditions and experiments.

#### Illusion strength measurement

As stated in the preregistration, one of our goals was to evaluate different possible methods of RHI strength measurement. First, we wanted to examine whether 1D solutions or particular dimensions of 2D configurations could be interpreted in terms of ownership. Such dimensions could be treated as internal ownership scales, allowing the calculation of “MDS ownership scores” for particular items (*i*) through normalization of point coordinates between 0 and 1, with the use of the formula

1 $${z}_{i}=\frac{{z}_{i}-min\left(x\right)}{max\left(x\right)-min\left(x\right)}$$

In averaged solutions, this normalized ownership score provided a general estimate of intensity of ownership feelings associated with particular items. For the purpose of illusion strength measurement, we wanted to select participants providing configurations defined by ownership-related dimensions. Then, we aimed to calculate illusion strength indices based on mean ownership scores associated with experimental items, and compare them between conditions using paired *t*-tests (note that for arm immobilization, the illusion strength index would be calculated by subtracting the ownership score from 1, reflecting the strength of the “ownership loss” experience).

The second variant of RHI strength measurement using MDS was based on the relative positions of experimental items with respect to the baseline items representing normal bodily feelings and empty glove (i.e., no ownership feelings). For RHI-based conditions, we aimed to calculate distances separating the rubber hand item from baseline items to obtain distance-based measures of “embodiment” (inversely proportional to the distance between the rubber hand item and the normal bodily feelings item) and “de-objectification” (proportional to the distance between the rubber hand item and the empty glove item). For the arm immobilization suggestion, these distance-based measures would indicate “de-embodiment” (proportional to the distance between the arm immobilization item and the normal bodily feelings item) and “objectification” (inversely proportional to the distance between the arm immobilization item and the empty glove item). In Experiment 2, we planned to compare (1) embodiment (synchronous RHI) with objectification (arm immobilization) indices as both measuring the effect in the direction of what an object “starts to be” (RHI: one’s body; arm immobilization: an external object) and (2) de-objectification (synchronous RHI) with de-embodiment (arm immobilization) indices as both measuring the effect in the direction of what an object “ceases to be” (RHI: an external object; arm immobilization: one’s body). Respective distances were compared using paired *t*-tests.

#### Familiarities

Our main research questions regarding both the bodily space structure and illusion strength measurement possibilities offered by inverse MDS were re-examined with the use of familiarity ratings as weights. Most likely, participants came to our lab having different experiences of fluctuations in body ownership—for example, some people might have never consumed alcohol, while others had been consuming it quite regularly. Individual differences in familiarity with given bodily experiences might have unequally impacted the reliability of dissimilarity estimations for certain items. To account for this possibility, we explored how condition-specific MDS configurations for bodily space change when mean familiarity ratings for particular items are used as weights, with higher weights assigned to more familiar experiences. For illusion strength measurement purposes, we carried out exploratory analyses related to how distance-based indicators of (de-)embodiment and (de-)objectification change when participant-specific familiarity ratings are used as weights in individual MDS solutions.

#### Data transformations

In order to calculate distance-based illusion (or suggestion) strength measures in 2D solutions, we used Procrustes alignment (Goodall, 1991; Rohlf & Slice, 1990) to transform pairs of MDS configurations obtained in different conditions to be aligned as closely as possible with respect to positions of homologous landmarks (i.e., the same items). In Procrustes alignment, one of the configurations is rotated, reflected, translated, and adjusted in size to optimally match the target configuration while preserving its original structure. Such a transformation eliminates all incidental or meaningless differences between the configurations, leaving only differences that were actually caused by the proximity data (Borg et al., 2018). This allows the calculation of Euclidean distances separating individual points within a shared configuration and comparison of these distances between two aligned configurations.

To assess the stability of bodily space across conditions and experiments, we also calculated several measures of overall configurational similarity for each Procrustes alignment we conducted, including the congruence coefficient of corresponding distances, correlation of the coordinates of corresponding points, and alienation coefficient (Mair et al., 2022). All these measures were benchmarked against 500 simulated fits of random configurations using the Procrustes test as proposed by Borg and colleagues (2018, p. 85). Finally, to assess the consistency with which individual items were located in the bodily space, we calculated pairwise distances between homologous landmarks in two aligned configurations.

### Hypotheses

The second variant of the proposed MDS-based illusion strength measurement methods, related to calculation of distances between experimental items and baseline items, was by definition much more versatile, as it did not require additional selection of individual solutions for those including ownership dimensions. Therefore, in the preregistration document, we posed four directional hypotheses related to distances between experimental items and baseline items which could be calculated in all individual solutions regardless of their structure. In particular, we expected the following:

1. The distance between the RHI-related item and the normal body item would be shorter in the synchronous than the asynchronous stimulation condition (Experiment 1; “embodiment effect”).
2. The distance between the RHI-related item and the external object item would be larger in the synchronous than the asynchronous stimulation condition (Experiment 1; “de-objectification effect”).
3. The distance between the RHI-related item and the normal body item would be shorter than the distance between the suggestion-related item and the external object item (Experiment 2; “RHI embodiment effect” stronger than “arm immobilization objectification effect”).
4. The distance between the RHI-related item and the external object item would be larger than the distance between the suggestion-related item and the normal body item (Experiment 2; “RHI de-objectification effect” stronger than “arm immobilization de-embodiment effect”).

We aimed to examine our hypotheses only in 1D and 2D solutions due to our pilot observations (*N* = 12) that virtually all of the participants successfully reduced the ownership space to two or fewer dimensions, as reflected in stress values for individual 2D solutions (*M* = 0.098; *SD* = 0.064; *max* = 0.202; 11/12 participants provided solutions with stress lower than 0.2).

## Results

### Experiment 1

#### Diagnostics and assessment of MDS solutions

##### 1D solutions

For each of the 25 participants, we fitted 1,000 interval MDS solutions with random starting points and performed a permutation test on the lowest stress solution. All of the participants passed permutation tests (all *ps* < 0.003), suggesting that meaningful (nonrandom) 1D structures could be obtained from individual proximity data in all cases. However, only three out of 25 provided satisfactory solutions according to the rule of thumb (stress < 0.2) in both conditions. Mean stress significantly exceeded the assumed threshold both in synchronous (*M* = 0.321; *SD* = 0.072) and asynchronous (*M* = 0.303; *SD* = 0.084) conditions.

Given that relatively high stress values were observed, individual 1D solutions were additionally visually inspected for interpretability in terms of a homogeneous ownership dimension. Visual analysis revealed that 12/25 (48%) synchronous solutions and 9/25 (36%) asynchronous solutions could be tentatively interpreted in terms of such an “ownership scale.” These numbers were substantiated by an ad hoc analysis (not included in the preregistered analysis plan) showing that the normalized distance between the two baseline items (empty glove item and normal bodily feelings item) was higher than 0.8 in only 11/25 synchronous solutions (44%) and 9/25 asynchronous solutions (36%). Taken together, these results clearly show that, for most of the participants, psychological bodily space seems to contain more than one dimension and can hardly be reduced to a unidimensional ownership scale. Therefore, we decided to abandon the preregistered analysis plan for 1D solutions and not to analyze them any further.

##### 2D solutions

We carried out an analogous procedure to assess individual 2D fits. All of the participants passed the permutation test (all *ps* < 0.013), with the majority of them providing two satisfactory solutions according to the rule of thumb. Only four solutions could be deemed unsatisfactory (2/25 in the synchronous condition, 2/25 in the asynchronous condition), as they gave a stress value slightly higher than 0.2. These solutions were additionally visually inspected for indications of possible task misunderstanding. On that basis, we decided to exclude one participant who provided a solution without any consistent grouping of the items, and with some peculiarities present (e.g., with dead hand and normal body items being close together in space) in the synchronous condition. Altogether, 24/25 solutions in the synchronous condition and 25/25 solutions in the asynchronous condition were included in further analyses.

To find general solutions for the bodily space, we fitted 1,000 interval MDS solutions with random starting points to averaged dissimilarity matrices in both synchronous and asynchronous conditions. The lowest observed stress values were below the rule-based cutoff point (synchronous, 0.158; asynchronous, 0.122), and lower than all values observed in respective permutation tests. Next, we explored influential points for the general solution through the stress-per-point (SPP) analysis. The items with the largest SPP values were rubber hand (17.76%) and cold hands (14.07%) for the synchronous condition, and sitting cross-legged (16.59%) and swelling (15.81%) for the asynchronous condition. Given that we did not observe unacceptably high SPP values (and none of the items consistently yielded highest SPP values), we concluded that solutions were unlikely to benefit from point exclusions and decided to keep all of the items in the space.

**Fig. 3:**
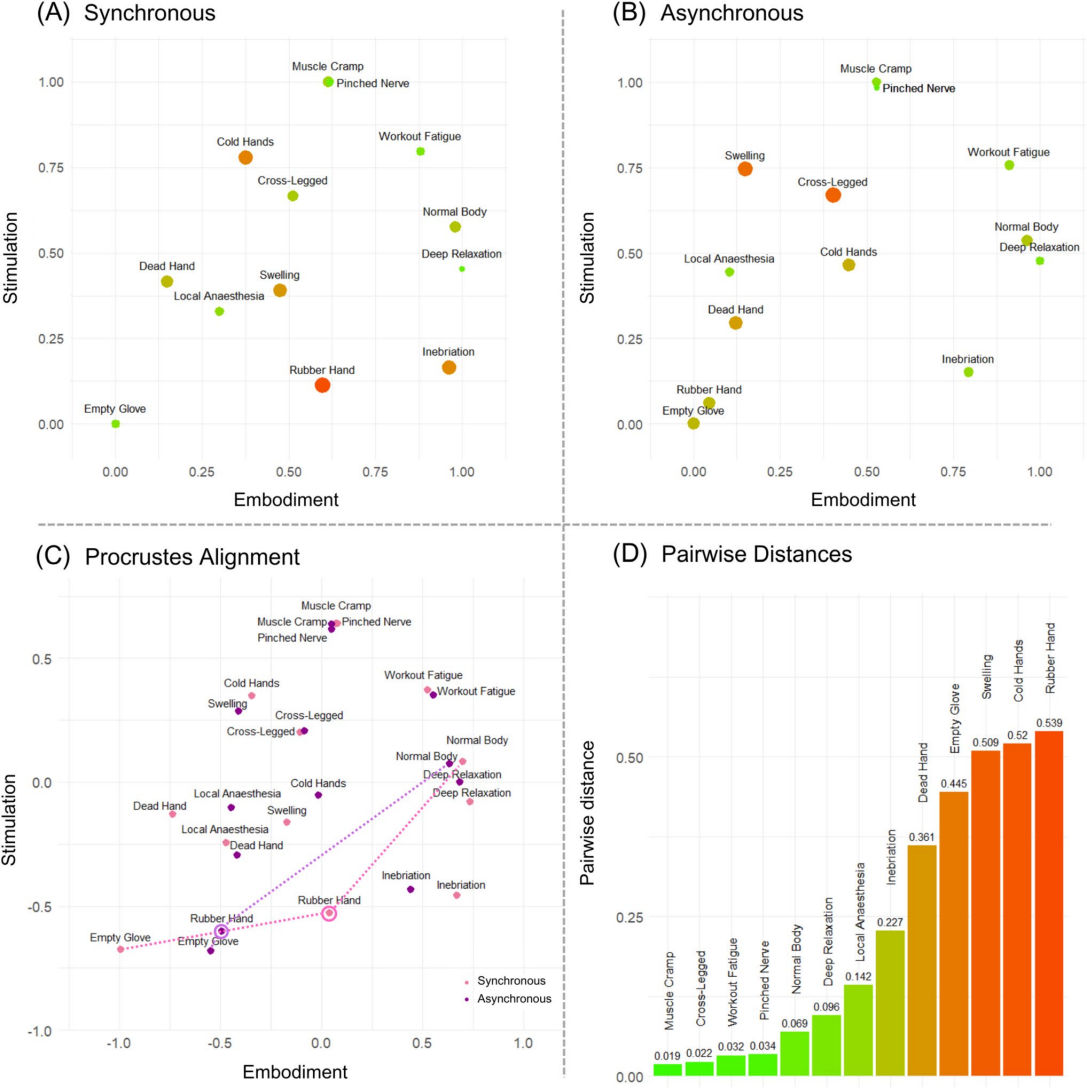
General 2D solutions for the bodily space in Experiment 1. *Note.* MDS solutions in both synchronous (**A**) and asynchronous (**B**) conditions were interpreted in terms of embodiment (X axis) and stimulation (Y axis) dimensions. Size and color of the points represent stress-per-point (SPP) values which inform about the degree to which particular items contributed to the misfit of the configurations, with larger reddish dots representing higher SPP values. When subjected to Procrustes alignment (**C**), solutions in synchronous (pink) and asynchronous (violet) conditions evidenced high structural similarity, suggesting that psychological bodily space is stable across conditions and can reliably be extracted from inverse MDS arrangements. Dashed lines present distances between the rubber hand item and baseline items in particular conditions. The largest pairwise distance was observed for the rubber hand item (**D**), indicating that this item changed its location between two configurations most substantially. Please note that, although we found no evidence for the rubber hand item being closer to the normal body item in the synchronous condition at the participant level, visual inspection suggests that it may be closer at the group level, as shown in (**C**)

Figure 3 presents the accepted solutions for the synchronous (Fig. 3A) and asynchronous (Fig. 3B) conditions. The configurations were rotated by 180° and 150°, respectively (cf. Mair et al., 2015). In contrast to developments in the body ownership field, which tend to describe bodily space in terms of correlated but independent dimensions of ownership feelings and sense of agency (Tsakiris et al., 2010), we interpreted two dimensions of our rotated solutions in terms of (1) embodiment (comprising both senses of ownership and agency) and (2) (intensity of bodily) stimulation. The latter dimension was interpreted with respect to the items associated with extreme values on the X axis (cf. Borg et al., 2018): lower values corresponded to experiences of stiffness, numbness, immobility, or relaxation (anesthesia, dead hand, inebriation, RHI), while higher values corresponded to experiences of tension or unusually intensive bodily stimulation (muscle cramp, pinched nerve, workout fatigue, swelling). When subjected to Procrustes transformation (Fig. 3C), general solutions in both conditions evidenced high configurational similarity. The congruence coefficient of corresponding distances (*c* = 0.964) and the correlation of the coordinates of corresponding points (*r* = 0.888) were both much higher than the highest values obtained in the Procrustes permutation test (*c* = 0.897; *r* = 0.657). The alienation coefficient (*a* = 0.265) turned out to be much lower than the minimum value observed in random simulations (*a* = 0.442). The analysis of pairwise distances (Fig. 3D) between homologous landmarks showed that the rubber hand item (*dist* = 0.539) changed its position between two configurations most substantially, which was expected as it was the only item that actually represented different experiences in two configurations (i.e., presumably, occurrence and absence of the illusion in synchronous and asynchronous conditions, respectively). However, it was closely followed by the cold hands (*dist* = 0.520) and swelling (*dist* = 0.509), which had switched locations between two configurations due to two local stress minima related to slightly different solutions**.** Notwithstanding the last finding, these results show high stability of the bodily space which was highly replicable across conditions.

#### Illusion strength measurement

Our diagnostic protocol for 2D solutions showed that these solutions reliably captured some nontrivial aspects of bodily space structure. Therefore, we decided to use these solutions for RHI strength measurement purposes. First, we calculated general estimates of the intensity of ownership feelings (MDS ownership score; *OS*_MDS_) in both conditions by normalizing coordinates on the embodiment dimension for the rubber hand item. The MDS ownership score turned out to be much higher (nominally) in the synchronous (*OS*_MDS_ = 0.598) than in the asynchronous condition (*OS*_MDS_ = 0.046).

**Fig. 4:**
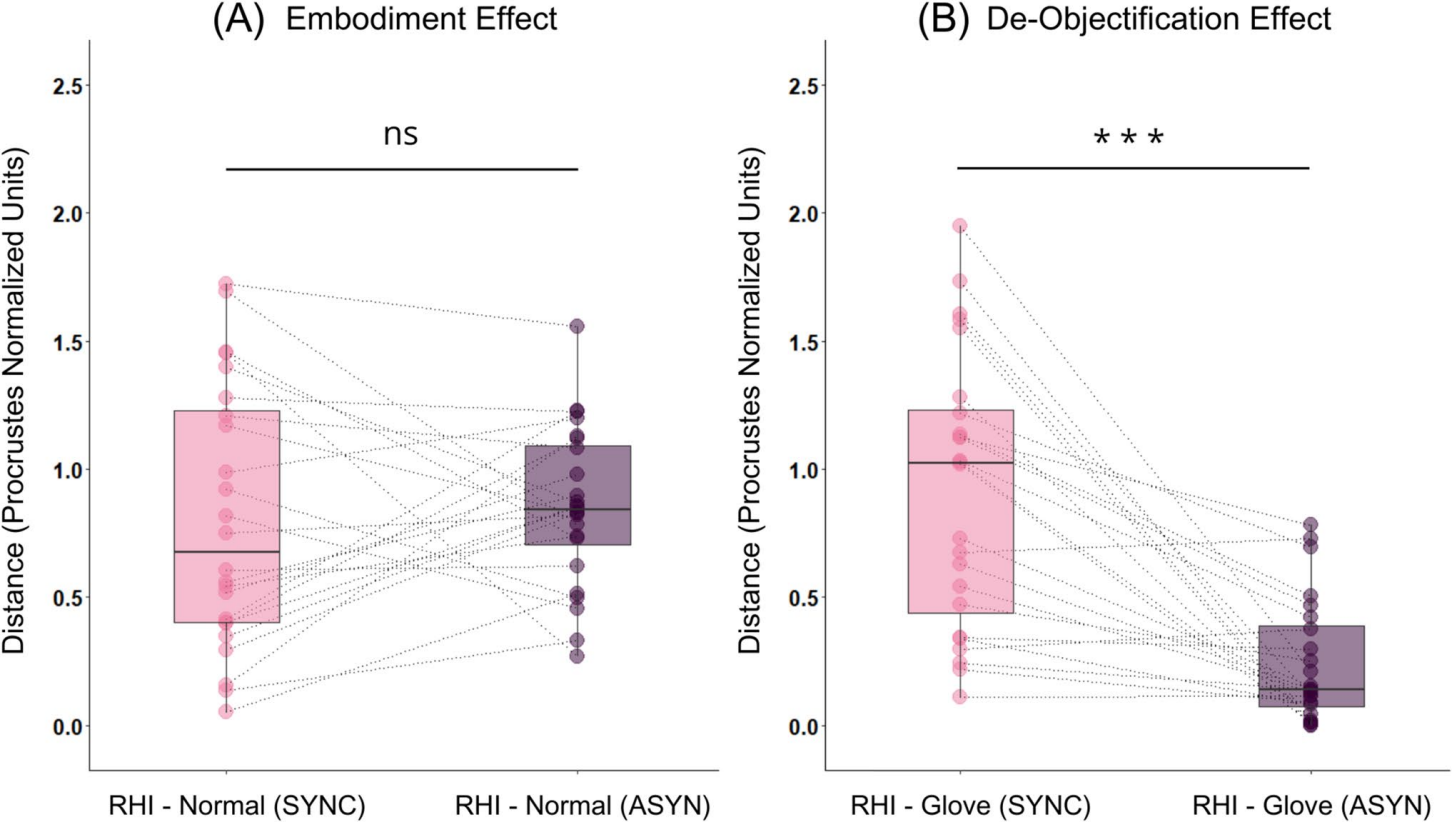
Comparison of Procrustes distances separating RHI-related items and baseline items in both synchronous (pink) and asynchronous (violet) MDS configurations. *Note.* No evidence for the embodiment effect was observed (**A**), as the rubber hand item was not significantly closer to the baseline item representing normal body ownership feelings in the synchronous than in the asynchronous condition. On the contrary, a strong de-objectification effect was found (**B**): in the synchronous condition, the rubber hand item was significantly farther from the empty glove item than in the asynchronous condition. Colored dots represent respective distances observed in individual solutions, with observations from the same participants connected with a dashed line. The distance is conveyed in Procrustes normalized units (Y axis). *** *p* < 0.001

To examine our hypotheses, we followed our preregistered analysis plan and compared mean distances between RHI-related items and baseline items in pairs of individual solutions aligned with the use of Procrustes alignment. We found no evidence for the embodiment effect; that is, the distance between the RHI-related item and the normal body item was not significantly shorter in the synchronous (*M* = 0.804; *SD* = 0.513) than in the asynchronous stimulation condition (*M* = 0.851; *SD* = 0.310), *t*(23) =  − 0.419; *p* = 0.340; *d* =  − 0.085 (Fig. 4A). On the other hand, a strong de-objectification effect was observed: the distance between the RHI-related item and the empty glove item was significantly larger in the synchronous (*M* = 0.917; *SD* = 0.536) than the asynchronous stimulation (*M* = 0.2421; *SD* = 0.2416) condition, *t*(23) = 5.528; *p* < 0.001; *d* = 1.128 (Fig. 4B). Moreover, while RHI-normal body distance was not significantly correlated with self-reported illusion strength as measured by questionnaires, *r*(22) =  − 0.239; *p* = 0.261, RHI-external object distance was, *r*(22) = 0.488; *p* = 0.016. This positive correlation coefficient means that the larger the distance between the rubber hand item and the empty glove item, the higher the strength of the illusion as reported with the use of a simple questionnaire.

For cross-validation, we also analyzed questionnaire scores, which yielded ratings similar to those obtained in other questionnaire-based studies (cf. Reader et al., 2021a), with self-reported RHI strength in the synchronous condition (*M* = 1.250; *SD* = 1.549) being significantly higher than in the asynchronous condition (*M* =  − 1.361; *SD* = 1.650), as shown by a one-tailed paired-samples *t*-test, *t*(23) = 6.576; *p* < 0.001;* d* = 1.342. The effect size was large, with Cohen’s *d* value slightly higher than for the de-objectification effect found using the MDS-based method. When normalized between the minimum and maximum values on the scale, mean questionnaire-based estimates for RHI strength were nominally higher in both synchronous (*OS*_Q_ = 0.708) and asynchronous (*OS*_Q_ = 0.273) conditions compared to the general MDS-based estimates (*OS*_MDS_ = 0.598 and *OS*_MDS_ = 0.046, respectively). These results show that MDS-based measurement estimated the RHI to be slightly weaker compared to questionnaire-based measurement.

### Experiment 2

#### Diagnostics and assessment of MDS solutions

##### 1D solutions

We followed an analogous procedure as in Experiment 1 to assess the 1D MDS solutions. Again, all participants passed respective permutation tests, but in each condition, only 1/27 participants provided solutions with stress values lower than 0.2. The mean stress of individual solutions crossed the rule-of-thumb threshold in both synchronous (*M* = 0.327; *SD* = 0.054) and immobilization (*M* = 0.333; *SD* = 0.069) conditions. Therefore, we decided to abandon the initial analysis plan for 1D solutions in Experiment 2 as well.

##### 2D solutions

Next, we evaluated individual 2D fits. Again, all participants passed the permutation test (all *p*s < 0.015). The mean stress of individual 2D solutions was below the rule-of-thumb threshold in both synchronous (*M* = 0.145; *SD* = 0.038) and immobilization (*M* = 0.147; *SD* = 0.047) conditions. Only four solutions were deemed unsatisfactory according to the 0.2 cutoff point (1/27 in the synchronous condition, 3/27 in the immobilization condition). These solutions were plotted and additionally visually examined. On that basis, we decided to exclude one participant who provided two solutions with no consistent grouping and some peculiarities present (e.g., with the dead hand and deep relaxation items being close together in the synchronous solution). Altogether, 26 participants were included in further analyses.

**Fig. 5:**
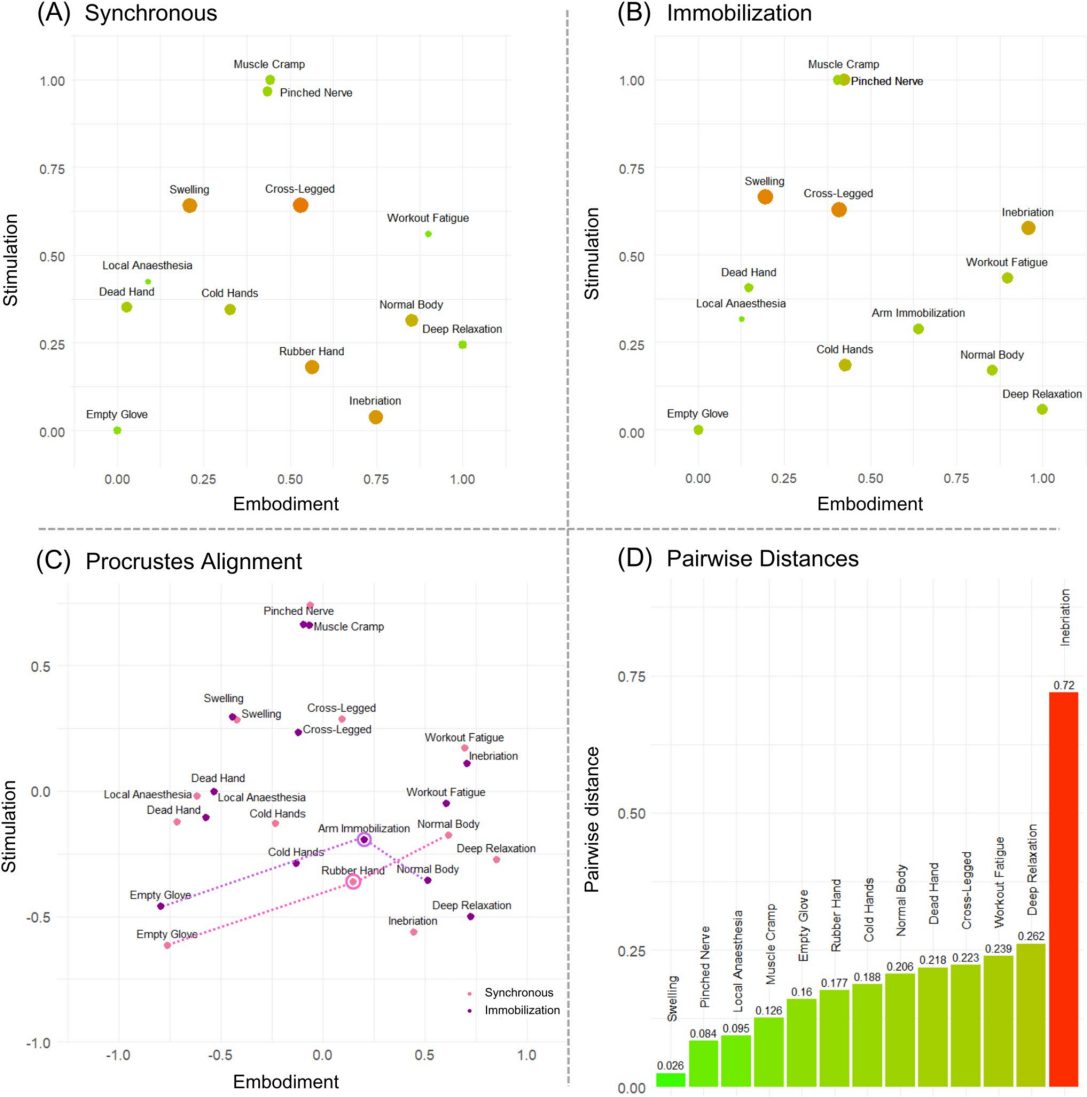
General 2D solutions for the bodily space in Experiment 2. *Note.* MDS solutions in both synchronous (**A**) and immobilization (**B**) conditions could again be interpreted in terms of embodiment (X axis) and stimulation (Y axis) dimensions. The size and color of the points represent stress-per-point (SPP) values which inform about the degree to which particular items contributed to the misfit of the configurations, with larger reddish dots representing higher SPP values. When subjected to Procrustes alignment (**C**), solutions in synchronous (pink) and immobilization (violet) conditions evidenced high structural similarity. Dashed lines present distances between experimental items and baseline items in particular conditions. The largest pairwise distance was observed for the Inebriation item (**D**), with the experimental item (representing either the rubber hand or the immobilized arm) occupying relatively similar locations in two configurations

A total of 1,000 interval MDS solutions with random starting points were fitted to averaged dissimilarity matrices in both the synchronous and immobilization conditions. The lowest observed stress values were similar to Experiment 1 (synchronous, 0.163; immobilization, 0.146) (Fig. 5), and lower than all values observed in respective permutation tests. Stress-per-point analysis of individual points in space showed that the largest SPP values were associated with sitting cross-legged (14.83%) and swelling (12.72%) for the synchronous condition or swelling (13.83%) and sitting cross-legged (13.55%) for the asynchronous condition. Even though the same two items contributed the most to the misfit of configurations in both conditions, their SPP values were relatively low. Thus, none of the points was excluded from the bodily space, and general solutions were accepted both for the synchronous and the immobilization condition.

Figure 5 presents rotated solutions for the synchronous (Fig. 5A) and asynchronous (Fig. 5B) conditions (the two configurations were rotated by 65° and 335°, respectively). Configurations were very similar to the ones obtained in Experiment 1 (see *Exploratory analyses* below), with both dimensions again interpretable in terms of (1) embodiment and (2) (intensity of bodily) stimulation. Interestingly, the baseline item representing a canonical case of full-body ownership (i.e., normal body) was not associated with the highest value on the sense of ownership dimension in either configuration. This might suggest that, at least for some participants, there are bodily experiences that might entail feelings of body ownership which are stronger than usual, for example through enhanced attention to or processing of bodily signals during workout or deep relaxation periods.

Again, when subjected to Procrustes alignment (Fig. 5C), general solutions in both conditions proved to be highly similar. Both the congruence coefficient of corresponding distances (*c* = 0.984) and correlation of the coordinates of corresponding points (*r* = 0.918) exceeded the highest values obtained in the Procrustes permutation test (*c* = 0.888; *r* = 0.657). The alienation coefficient (*a* = 0.177) was much lower than the minimum value observed in random simulations (*a* = 0.442). The analysis of pairwise distances between corresponding points in the two configurations showed that the only point that substantially changed its position between the configurations was the inebriation item (*dist* = 0.720). Surprisingly, items related to experimentally elicited experience (RHI or suggestion) were relatively close together in space after the alignment (*dist* = 0.177), suggesting that bodily experiences related to RHI and arm immobilization might actually be similar, even though the two illusory experiences work in different directions.

#### Illusion strength measurement

General estimates of intensity of ownership feelings (MDS ownership scores; *OS*_MDS_) were calculated in both conditions. The RHI was again associated with moderate levels of ownership transfer toward the rubber hand, as reflected in the respective MDS ownership score (*OS*_MDS_ = 0.563). The MDS-based score for the arm immobilization condition indicated that, during the suggestion, perceived ownership over one’s real hand was lower to some extent (*OS*_MDS_ = 0.641). Assuming the maximum normalized ownership score as baseline, the MDS ownership score for the arm immobilization suggestion translates to the MDS ownership loss index—reflecting the actual strength of the suggestion—equal to 1 − 0.641 = 0.359. However, if we take the normalized ownership score for the normal body item (*OS*_MDS_ = 0.846) as the true baseline, we obtain an MDS ownership loss index equal to (0.846 − 0.641) × (1/0.846) = 0.242.

**Fig. 6:**
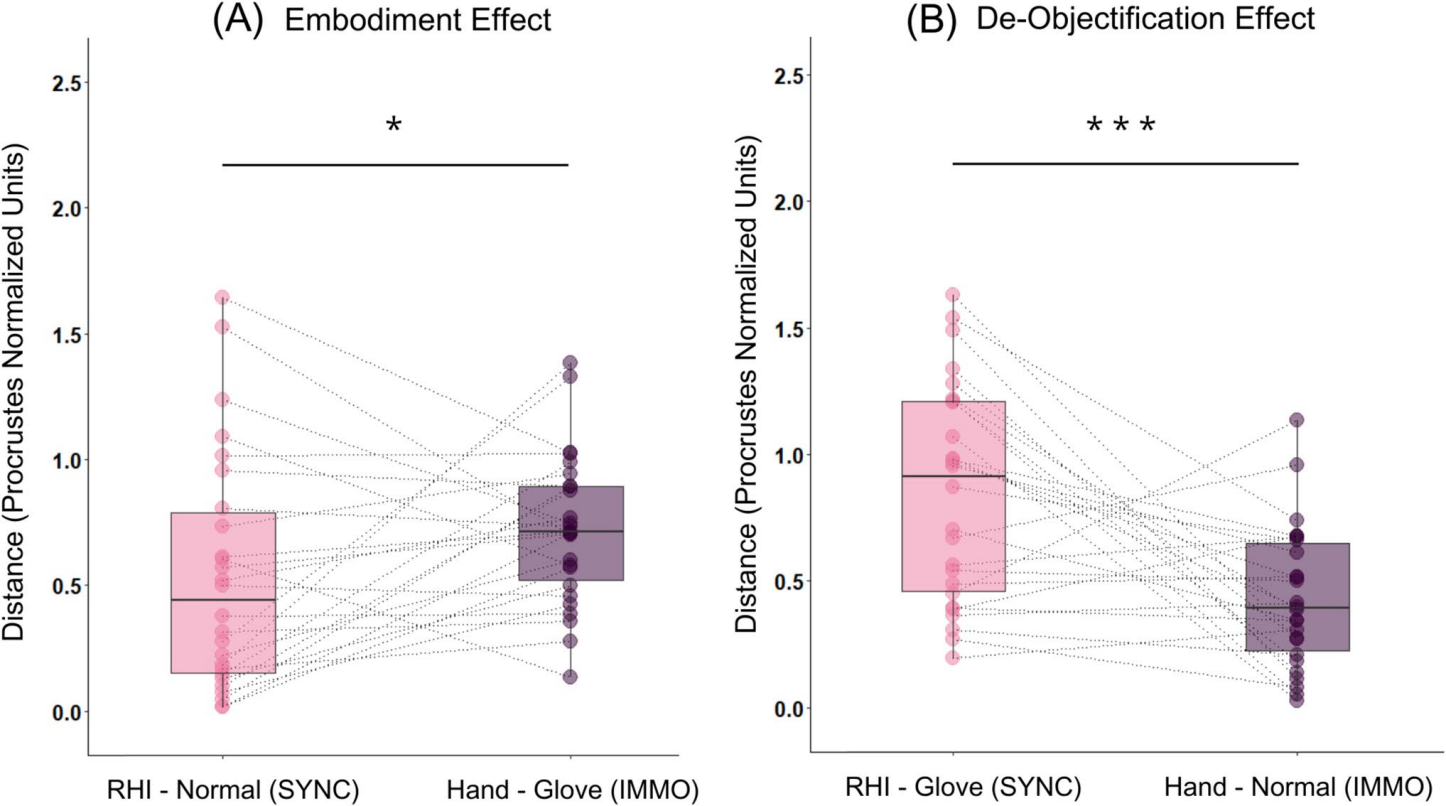
Comparison of Procrustes distances separating experimental items and baseline items in both synchronous (pink) and immobilization (violet) MDS configurations. *Note.* In Experiment 2, the embodiment effect was observed (**A**), as the distance separating the rubber hand item and the normal bodily feelings item was shorter than the distance between the arm immobilization item and the empty glove item. However, the de-objectification effect (**B**) was again found to be more pronounced. Colored dots represent respective distances observed in individual solutions, with observations from the same participants connected with a dashed line. The distance is conveyed in Procrustes normalized units (Y axis). * *p* < 0.05; *** *p* < 0.001

Finally, we followed our preregistered analysis plan to examine our hypotheses. Because of the different directions in which RHI and arm immobilization suggestion work (i.e., body ownership gain in RHI vs. body ownership loss in the suggestion), we compared pairs of distances (1) in the direction of what an object “starts to be” (i.e., rubber hand − normal body distance vs. immobilized arm − empty glove distance) and (2) in the direction of what an object “ceases to be” (i.e., rubber hand − empty glove distance vs. immobilized arm − normal body distance). The results showed that the distance between the RHI-related item and the normal body item (*M* = 0.529; *SD* = 0.466) was significantly shorter than the distance between the arm immobilization item and the empty glove item (*M* = 0.713; *SD* = 0.298), *t*(25) =  − 1.817; *p* = 0.041; *d* =  − 0.356 (Fig. 6A), suggesting that embodiment of the rubber hand was more vivid than objectification of one’s own hand during the suggestion. Additionally, there was a strong effect in the direction of what an object “ceases to be”: The distance between the RHI-related item and the empty glove item (*M* = 0.857; *SD* = 0.434) was significantly larger than the distance between arm immobilization item and the normal body item (*M* = 0.436; *SD* = 0.283), *t*(25) = 3.742; *p* < 0.001; *d* = 0.734 (Fig. 6B). These results provide substantial evidence that the de-objectification of the rubber hand was much stronger than the objectification of one’s own hand during the suggestion. In the RHI condition, we found significant correlations between MDS-based measures and questionnaire measures, both for RHI-normal body distance, *r*(24) =  − 0.605, *p* = 0.001, and for RHI − empty glove distance, *r*(24) = 0.624, *p* < 0.001. In the arm immobilization condition, the correlation was significant only for the arm immobilization − empty glove distance, *r*(24) =  − 0.610, *p* < 0.001. The correlation between the arm immobilization − normal body distance and questionnaire-based subjective strength of the suggestion did not reach the statistical significance threshold, *r*(24) = 0.356, *p* = 0.075.

Questionnaire-based measurement was again sensitive to differences between conditions, with self-reported RHI strength in the synchronous condition (*M* = 1.538; *SD* = 1.558) being significantly higher than disownership in the arm immobilization condition (*M* =  − 1.603; *SD* = 1.506), as shown by a one-tailed paired-samples *t*-test, *t*(25) = 8.181; *p* < 0.001;* d* = 1.604. The effect size was very large, with Cohen’s *d* visibly exceeding (approximately twice as large as) the effect size estimate for de-objectification. When normalized between the minimum and maximum values on the scale, mean questionnaire-based estimates for RHI strength (*OS*_Q_ = 0.756) were again higher than the MDS-based estimate (*OS*_MDS_ = 0.563), while estimates of arm immobilization strength were similar for both methods (*OS*_Q_ = 0.233; *OS*_MDS_ = 0.242). These results confirm our previous finding that the MDS-based method yields lower ownership estimates in the synchronous condition than questionnaires.

### Exploratory analyses

Our planned analyses showed that general MDS configurations for the bodily space were highly stable across conditions. Here, we decided to compare MDS solutions from Experiment 1 and Experiment 2 to explore whether bodily space structure is also stable across separate experiments including different participants. For this purpose, we performed the Procrustes alignment on MDS solutions observed in the synchronous RHI condition in both experiments. Again, we observed high structural similarity between two spaces, as evidenced by high congruence coefficient values for the corresponding distances (*c* = 0.972) and correlation of the coordinates of corresponding points (*r* = 0.915), and low alienation coefficient values (*a* = 0.235). The analysis of pairwise distances between homologous landmarks in two configurations revealed that it was the cold hands (*dist* = 0.508) and swelling (*dist* = 0.450) items that most substantially changed location in the bodily space between two experiments. The rubber hand item was associated with the fourth-largest distance (*dist* = 0.241). These results suggest that bodily experiences associated with RHI are consistently located in the bodily space, demonstrating the validity of the MDS-based measurement of RHI strength.

Given the highly correlated MDS configurations between synchronous RHI conditions in Experiment 1 and Experiment 2, we decided to aggregate all 50 participants who were included in previous analyses to provide the most robust and generalizable 2D solution for the bodily space. These final analyses were performed both with and without mean familiarity ratings for particular items used as weights. The solutions were rotated by 40° (unweighted) and 100° (weighted). Once again, we reproduced the two-dimensional bodily space structure with principal dimensions interpretable in terms of magnitude of embodiment and amount of stimulation. For the unweighted solution, the normalized ownership score for the rubber hand indicated moderate strength of ownership feelings (*OS*_MDS_ = 0.579) and low felt stimulation (*SS*_MDS_ = 0.227). The weighted solution also provided scores which lend the interpretation of RHI as related to moderate feelings of ownership and low perceived stimulation (*OS*_MDS_ = 0.585; *SS*_MDS_ = 0.046).

**Fig. 7:**
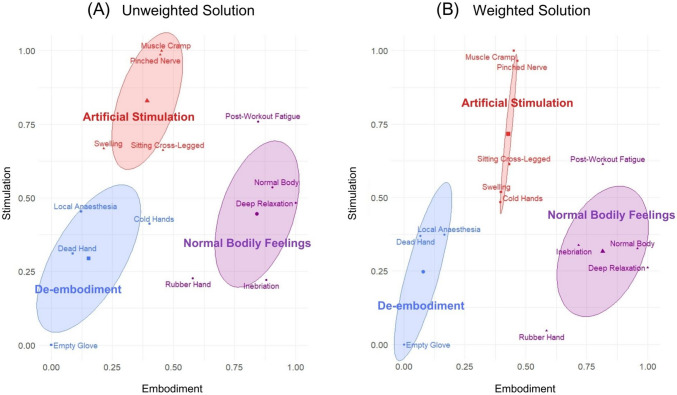
Three clusters in the bodily space on data aggregated across synchronous RHI conditions in Experiments 1 and 2. *Note.* Three clusters, related to the feelings of de-embodiment (blue), artificial stimulation (red), and normal bodily feelings (purple), were determined with the use of the *k*-means method on unweighted (**A**) and weighted (**B**) solutions. In both cases, feelings over the rubber hand during RHI were included in the normal bodily feelings cluster. Colored ellipses represent 95% confidence intervals for respective cluster means

A clustering analysis using the *k*-means method was carried out on solutions based on aggregated data from Experiments 1 and 2. For both weighted and unweighted solutions, NbClust indicated that three clusters was the optimal number. The analysis revealed that the bodily space can be organized into de-embodiment, artificial stimulation, and normal bodily feelings clusters (Fig. 7). As evidenced by cluster means, de-embodiment was characterized by low perceived embodiment (unweighted 0.153; weighted 0.078) and bodily stimulation (unweighted 0.294; weighted 0.248), artificial stimulation was associated with moderately intense feelings of embodiment (unweighted 0.392; weighted 0.429) and high perceived stimulation (unweighted 0.829; weighted 0.716), and the normal bodily feelings cluster was related to strong perceived embodiment (unweighted 0.842; weighted 0.815) and moderate stimulation (unweighted 0.445; weighted 0.317). The only difference between the unweighted (71.97% of variance explained) and weighted (71.74% of variance explained) clustering solutions was the cold hands item, which was included in the de-embodiment cluster in the former, and the artificial stimulation cluster in the latter. RHI was assigned to the normal bodily feelings cluster in both weighted and unweighted solutions, although its ownership score was the lowest of all items in this cluster.

Finally, we decided to rerun the analyses related to our preregistered hypotheses using individual solutions weighted by familiarity ratings provided by respective participants. All 52 participants were included in familiarity-weighted analyses. For three participants whose familiarity data were missing, mean familiarity ratings observed in Experiment 1 were used as weights. We found that weighting items by familiarity does not seem to improve the sensitivity of RHI strength measurement. An analogous pattern of results was observed: We found no evidence that the distance between the rubber hand item and the normal body item was shorter in the synchronous (*M* = 0.079; *SD* = 0.043) than the asynchronous stimulation condition (*M* = 0.090; *SD* = 0.030), *t*(24) =  − 1.099; *p* = 0.141; *d* =  − 0.220. For weighted MDS configurations, neither the rubber hand − normal body distance, *r*(24) =  − 0.248, *p* = 0.232, nor the rubber hand − empty glove distance, *r*(24) = 0.386, *p* = 0.057, correlated significantly with questionnaire-based estimations of illusion strength in the synchronous condition. In Experiment 2, familiarity-weighted analyses seemed to even slightly reduce sensitivity, as they failed to provide decisive evidence that the distance between the rubber hand item and the normal body item (*M* = 0.055; *SD* = 0.045) was significantly shorter than the distance between the arm immobilization item and the empty glove item (*M* = 0.072; *SD* = 0.029), *t*(26) =  − 1.541; *p* = 0.068; *d* =  − 0.296.

## Discussion

In the current paper, we introduced a novel method of self-reported RHI strength measurement, in which we used the inverse MDS methodology (Kriegeskorte & Mur, 2012) to assess the degree of perceived similarity between RHI and other bodily experiences known from everyday life. Participants were asked to repeatedly arrange 13 items representing various experiences differing in terms of associated bodily sensations—including the rubber hand item representing illusory ownership feelings during RHI—so that distances between items directly translated to perceived dissimilarity between experiences. Proximity data obtained from these arrangements were then represented as distances between points in a multidimensional space. This allowed us to determine the structure of this bodily space as well as approximate felt ownership over the rubber hand during RHI. Importantly, this MDS-based approach caused the response space to be constrained by the phenomenological context, since participants could not arbitrarily adjust the position of the rubber hand item without disrupting all relations of this item in the bodily space.

Across two experiments, we have shown that our novel MDS-based approach is a promising method for RHI strength measurement. It is sensitive to differences between typically used experimental (i.e., spatiotemporally congruent visuo-tactile stimulation) and control conditions (i.e., asynchronous visuo-tactile stimulation) (Experiment 1) as well as differences between RHI and hand-related suggestions (Experiment 2). Normalized ownership scores yielded by our method as well as the clustering analysis suggested that RHI is experienced in a way that resembles regular bodily feelings, although the intensity of perceived ownership seems to be attenuated compared to regular bodily perception. In the context of our particular study, the method provided lower estimates of the strength of ownership feelings during the RHI than questionnaire-based self-report measures.

In Experiment 1, we found no evidence that the rubber hand felt more like one’s own body in the synchronous condition than in the asynchronous condition. This contradicts a large body of questionnaire-based, behavioral, and neurophysiological studies (Ehrsson, 2020). While we do not interpret this finding as undermining the authenticity of ownership feelings in the RHI, it may have important implications. First, our study focused on similarities between subjective bodily experiences, and embodiment of the rubber hand may qualitatively differ from everyday ownership, as the hand remains immobile and receives very specific stimulation. MDS ownership scores obtained in the current study suggest that experienced ownership of the rubber hand is partial (Exp. 1, 56.3%; Exp. 2, 59.8%). These values converge with previous research using self-report measures, such as questionnaires (median responses consistently on the fifth point of a seven-point scale—equivalent to 66.6% of the scale range—across all analyzed datasets; Reader et al., 2021a, Fig. 1) or ownership potentiometers (55.3%; Finotti et al., 2023). This converging evidence indicates that the common assumption that ownership feelings during the RHI closely resemble everyday ownership may need to be reconsidered. An alternative explanation is that differentiating the rubber hand from an external object may feel more natural as a way of expressing one’s experience during the illusion, particularly because an external object provides a firmly anchored baseline representing no ownership. Arguably, it is impossible to feel less ownership than over an external object. In contrast, experiences of greater-than-usual embodiment are in principle possible—for example, during deep relaxation, as also suggested by MDS configurations in Experiment 2. The observation that the canonical case of normal bodily ownership appears less stable in psychological bodily space may also account for lower reliability of distance-based measures that rely on this baseline case.

An important strength of our MDS-based approach is that it enables the determination of psychological bodily space in a data-driven, bottom-up manner. The MDS solutions observed in our study proved to be highly structurally similar both across conditions and across experiments conducted on different samples. These solutions provided revelatory insights into the structure of subjective bodily space: we showed that it can be reduced to two principal dimensions—embodiment and stimulation intensity—regardless of the fitting procedure applied. When individual experiences were clustered based on their coordinates in this two-dimensional space, three main categories of bodily experiences emerged: de-embodiment (weak feelings of both embodiment and stimulation), artificial stimulation (intermediate feelings of embodiment coupled with high perceived stimulation), and normal bodily feelings (strong embodiment with moderate feelings of stimulation). To the best of our knowledge, no previous account has proposed such a conceptualization of how bodily experiences are mentally represented, as prior work has typically drawn upon the distinction between ownership and agency (Longo & Haggard, 2012). Nonetheless, there are points of convergence with earlier findings. First, in both psychometric studies examining the multifaceted phenomenology of the RHI, ownership and agency appeared as subcomponents of the same overarching embodiment factor (Longo et al., 2008) or were not distinguished at all (Romano et al., 2021). These results are consistent with our finding that ownership and agency collapsed into a single embodiment dimension, suggesting that although ownership and agency do not share neural representations (Abdulkarim et al., 2023; Tsakiris et al., 2010) and can be experimentally dissociated (Kalckert & Ehrsson, 2012), they may not be readily distinguished in participants’ mental representations of bodily space. Second, the low sensory stimulation associated with the RHI in our study may correspond to the attenuation of tactile and somatosensory signals reported in both behavioral (Ataka et al., 2022; Rossi Sebastiano et al., 2022) and neurophysiological research (Sakamoto & Ifuku, 2021; Zeller et al., 2016). Nevertheless, it should be noted that the issue of somatosensory attenuation during the RHI remains debated, as direct electrophysiological recordings in the hand area of the primary somatosensory cortex have not revealed differences in touch-evoked responses between ownership (synchronous) and no-ownership (asynchronous) conditions (Guterstam et al., 2019).

However, even though almost all participants provided satisfactory 2D solutions, it does not follow that psychological bodily space is strictly two-dimensional. Three- and four-dimensional solutions entailed further substantial reductions in the stress parameter and could likely provide a more comprehensive description of the bodily space, in line with psychometric research on RHI (Longo et al., 2008; Romano et al., 2021). Because the structure of bodily space depends on the set of items included, the present study does not offer a comprehensive assessment, and our proposed description should be regarded as preliminary. The choice of items in our study was primarily guided by the need to anchor RHI assessments within familiar experiences of ownership fluctuations. To this end, we drew on previous qualitative interviews (Bartoletti et al., 2023; Lewis & Lloyd, 2010; Valenzuela Moguillansky et al., 2013; also Longo et al., 2008, in a broader psychometric context) that reported spontaneous reminiscences of unusual ownership feelings experienced during the RHI. These reports leaned more heavily toward experiences of disturbed ownership and agency and peculiar sensations; however, they may have overlooked possible dissociations between ownership and referral of touch (Tosi et al., 2024) or between ownership and self-location (Serino et al., 2013), which rarely diverge in nonclinical populations. Future studies aimed at further exploring the dimensions of bodily space—especially in clinical populations—should include as many items as feasible. The list of items used in the present study is not exhaustive and could easily be extended, while certain items might be removed as they proved only weakly informative (e.g., swelling and sitting cross-legged).

While our results provide valuable insights into RHI and the bodily space, the study also has important limitations. First, since MDS cannot insert semantic information into bodily space solutions, interpretations of dimensions and clusters fall to the expert researcher. As such, they are inherently arbitrary. This becomes problematic when we consider that point coordinates along specified dimensions—which directly translate to normalized scores for particular items and the clusters they belong to—depend on the specific solutions and rotation values. Since multiple local stress minima related to slightly different solutions exist, items might change locations or even “switch places” between conditions, as occurred with the swelling and cold hands items in Experiment 1 (Fig. 3A, B). As a result, in the synchronous condition of Experiment 1, cold hands was counterintuitively associated with relatively high stimulation, while swelling was associated with moderate stimulation, contrary to all other solutions in this study. In line with our preregistration, we chose the lowest stress solution; however, an alternative approach focused on interpretability exists. For example, Borg et al., (2018, p. 79) note that if “(…) there exist several different solutions that all have almost the same small Stress value, (…) the user can pick the solution that is most convincing in terms of interpretation.” Indeed, in our study we found solutions for the synchronous condition with similarly low stress values that would “correctly” associate the cold hands experience with moderate stimulation. For future studies, researchers might consider adopting this more theoretically involved approach focused on maximum interpretability, acknowledging that normalized dimension scores should be interpreted as approximate.

Another important limitation relates to other arbitrary choices in the MDS procedure. As noted above, the final solution for the psychological space of interest depends on the choice and number of items. In our study, certain classes of experiences—for example, those related to sensory attenuation or diminished agency—may have been overrepresented, potentially promoting the observed two-dimensional solutions. The proposed bodily space therefore requires further validation with a wider set of bodily experiences; however, it is essential that all items represent events likely to have been experienced by most participants. Additionally, participants’ solutions might perhaps be influenced by the selection of images representing particular experiences. Our images might have affected individual arrangements due to idiosyncratic associations either increasing or decreasing perceived similarities between pairs of items. While we do not see clear reasons why such biases would be systematic, it remains possible. To assess the robustness of the MDS-based method, future studies could use alternative image sets to determine whether solutions for the bodily space remain consistent.

The final limitation concerns the use of questionnaires and the MDS-based method within the same study. When introducing the MDS-based method, we decided to include questionnaire assessments for purposes of cross-method comparison and validation. However, this meant that participants were exposed to ownership statements before performing the MDS arrangements. These items may have drawn attention to the issue of body ownership and thereby introduced bias or shaped participants’ expectations. Future studies seeking further empirical comparisons between RHI questionnaires and MDS would likely benefit from administering the two methods on separate laboratory days. This design would have allowed for a more meaningful exploration of potential relationships or correlations across the different measures.

We believe that the present method can be used alongside questionnaires as a complementary tool for RHI strength self-assessment. Based on the results of our study, we can offer some methodological advice for future studies using the proposed method strictly for RHI strength measurement purposes. Out of two possible means of measurement offered by the current method, measurement in the direction of what an object “ceases to be” (e.g., a rubber hand ceases to be an object) is more sensitive to differences between conditions than measuring the effect in the direction of what an object “starts to be” (e.g., a rubber hand starts to be one’s body). In both experiments, analyses based on calculating distances between the rubber hand item and the empty glove item differentiated the RHI from control conditions much more clearly. Given the strong and robust effect of the primacy of the de-objectification over embodiment direction in RHI measurement, future studies should use the former to obtain the de-objectification index as an indirect proxy of embodiment, to be compared between the conditions of interest. We also suggest that researchers refrain from RHI measurement based on 1D solutions or unidimensional ownership scales, as they necessarily rely on individual participants providing solutions with dimensions interpretable in terms of embodiment. In MDS, it is common for the individual solutions to deviate from the averaged group-level solution (Borg et al., 2018), which was also observed in the current study. Our participants provided a wide variety of solutions, from those resembling a general bodily space structure, through circular arrangements centered around a particular item, to those grouping items into a few categorical clusters. However, we perceive this to be more a feature than a bug, reflecting the minimal amount of suggestion and unconstrained character of the method. Any problems that this interindividual variability might cause can be avoided by measuring relative distances from baseline items, which can be done regardless of the structure of individual configurations.

Finally, our MDS-based method opens up exciting new possibilities for measuring the strength of bodily illusions. Most importantly, the method might capture inherent differences between various phenomena which are now gathered under the umbrella term of *body ownership transfer illusions*, but might actually include qualitatively different experiences. These phenomena include, among others, “invisible body” illusions elicited by spatiotemporally congruent tactile stimulation of observed empty space (Guterstam et al., 2013), somatic RHI (Ehrsson et al., 2005), illusions allowing bodily transfer to an external object through motor control, in both real (Kalckert & Ehrsson, 2012) and virtual environments (Sanchez-Vives et al., 2010), or “full-body” illusions including splitting of one’s body location and the origin of first-person visual perspective (Ehrsson, 2007; Lenggenhager et al., 2007). More fine-grained or multifaceted differences in phenomenology between these experiences may currently be lost, as they are typically assessed using similar variants of Likert-type ownership scales. The MDS-based method could also be applied to re-examine findings in the literature that do not present a consistent picture of the relationships between the RHI and demographic variables, individual differences, or comorbid conditions. For example, RHI strength has been reported as attenuated with age (Ferracci & Brancucci, 2019; Kállai et al., 2017), as higher in both younger and older adults compared to middle-aged adults (Marotta et al., 2018), or as unrelated to age altogether (Campos et al., 2018; Palomo et al., 2018). As a result, we still know relatively little about the relation between age and susceptibility to body-transfer illusions. MDS-based measurement may provide an additional angle for addressing research questions that have remained unresolved over the years.

## Conclusions

Using an approach grounded in the theory of illusion strength measurement, we developed a novel self-report measure of RHI based on inverse multidimensional scaling. This measure proved reliable, yielding consistent bodily space solutions both within participants and across experiments and conditions. MDS-based assessment of RHI strength differentiated between experimental and control conditions but may be more conservative than traditionally used questionnaires; however, further validation is needed to clarify its sensitivity and specificity relative to questionnaire-based measures. The RHI was characterized by a moderately high degree of embodiment and low amount of perceived stimulation, and was clustered together with experiences associated with normal feelings of body ownership. We believe that the proposed method can be widely adopted in body ownership research as a complementary self-assessment tool, as it is relatively easy to implement and might entail additional benefits related to the validity of measurement.

## Supplementary Information

Below is the link to the electronic supplementary material.

Supplementary file1 (PDF 225 kb)

## Data Availability

All research materials (including supplementary materials), databases, and R files (including scripts for data preparation and exclusions, models, visualizations, and analysis pipeline) are available at the Open Science Framework repository https://osf.io/rtpj8. All hypotheses as well as the data analysis plan had been preregistered at OSF in a time-stamped document (https://osf.io/amzk4) before the data collection started.

## Code availability

R files including scripts for data preparation and exclusions, models, visualizations, and analysis pipeline are available at https://osf.io/rtpj8.
