# Supplementary material for: Novel method for rubber hand illusion strength measurement based on inverse multidimensional scaling
Source: Behav Res Methods. 2025 Dec 2;58(1):15. doi: 10.3758/s13428-025-02900-2 (PMC12672668; doi:10.3758/s13428-025-02900-2)
Supplement: Supplementary file 1 — Supplementary file1 (PDF 225 kb) [file 13428_2025_2900_MOESM1_ESM.pdf]

Supplementary Material S1 - Item Choice

(1) **Cold Hands**

Bartoletti et al., 2023: *"Some unpleasant sensations were widely shared by participants such as the feeling that the hidden hand was cold or numb."*

(2) **"Dead" Hand**

Valenzuela-Moguillansky et al., 2013: *"I feel like when your hand is numbed, when you fall asleep on your hand and you have to take it and move it around"*

(3) **Sitting Cross-Legged**

Bartoletti et al., 2023: *"It's like when you're sitting cross-legged, and you can't feel your legs."*

(4) **Deep Relaxation**

Bartoletti et al., 2023: *"It's relaxing, especially when it's always the same thing because there's no repetition and there's less stimuli, there's no alternation so it's less stimulating, and so it's relaxing"*

(5) **Swelling**

Valenzuela-Moguillansky et al., 2013: *"What I feel now is as if this [the rubber hand] was my hand, but as if it was a little bit swollen, I mean as if a wasp had bitten me (...) it's crazy"*

(6) **Inebriation**

Bartoletti et al., 2023: *"Always the impression of being drunk. When you were touching the index finger of the fake hand, I really felt like you were touching my index finger."*

(7) **Workout Fatigue**

Valenzuela-Moguillansky et al., 2013: *"I see that the hand does not move, I have the*

For Review Only

feeling that it does not answer. (...) I feel my arm like a dead weight, something very heavy."

(8) **Pinched Nerve**

Lewis & Lloyd, 2010: "*It's a really strange feeling, kind of tingly almost…I found moving it a bit difficult."*

(9) **Muscle Cramp**

Lewis & Lloyd, 2010: "*When participants are allowed to see their hand and move it again, they mostly describe it as cold, stiff, and initially difficult to move. It is as if the hand is slowly regaining function"*

(10) **Local Anaesthesia**

Lewis & Lloyd, 2010: "*It just feels numb like there's something wrong with my nerve or something"*

For Review Only

Supplementary Material S2 - Instructions

(1) ‘Welcome to the next part of the study, in which you will perform a short computer task. Your task will be to arrange 13 images representing different bodily experiences you might know from everyday life. Ten of these relate to different situations of unusual bodily experience—you are probably familiar with at least some of them, perhaps even all of them. The 11_{th} image relates to the natural perception of your body—as you normally feel it. The 12_{th} image refers to an external object—an empty glove that you do not perceive as part of your body and from which no sensations flow. The last, 13_{th} image refers to the way the rubber hand was experienced during the experiment. You have been given a *crib sheet* from the experimenter with descriptions for each picture—you can use it during the study. Press the spacebar to move forward with the instructions."

(2) ‘In this task we are only interested in sensations—we know that the rubber hand is not part of your body. We are only asking you to compare the sensations "flowing" from the rubber hand during the experiment to other bodily experiences which you may have previously experienced. People experience the rubber hand in different ways—you may perceive the experience as similar to any of the situations represented in the images, as well as to many at once or none at all. Any arrangement of the images is fine—just be as honest and accurate in your arrangements as possible. If this is not your first approach to the task, remember to only assess the experience you have just experienced, without taking previous experiences into account. Press the spacebar to see the exact instructions for the task.’

(3) ‘The task will be to arrange 13 items inside a white circular area. In your arrangements you should be governed only by subjectively perceived similarity in how you experience your body in these everyday situations. Similarity can refer to different aspects of experience, but

For Review Only

must strictly relate to sensations (e.g. tactile or flowing from inside the body), the perceived ability to move a particular body part, or a general "bodily vibe". Similar experiences should be placed close together (they can even overlap if you perceive them as very similar). Dissimilar experiences should be placed far apart.'

(4) 'Example: two elements placed close to each other—high similarity of experience. The elements have been randomly selected for the example— you may perceive them either as not similar or very similar.'

(5) 'Example: two elements placed far from each other—low similarity of experience. The elements have been randomly selected for the example—you may perceive them either as not similar or very similar.'

(6) 'Exact item positions don't matter much, only the relative positions between two items in a pair do—the smaller the distance between two items, the more similar they are to each other. Also, the exact way in which all items are arranged is not relevant—the arrangements can be anything. It is crucial, however, to make sure before eventually accepting the arrangement that distances in all item pairs are consistent with how you perceive similarities between them—with 13 items this is not easy, and in this task every pair matters! The most important rule is one: similar experiences must be close together, dissimilar ones far apart.'

(7) 'During the task you will be asked to make several arrangements of the same items—this is needed to accurately determine which experiences (and to what extent) you consider similar and dissimilar. For further arrangements, the programme may select and present a smaller number of items (e.g., 5)—in this case, you can think of the white area as zoomed in

For Review Only

and arrange the elements using all the available space. You will make a maximum of 12 arrangements—their exact number depends on the computer program. When the task is done, a relevant message will be displayed. '

(8) "Please remember that we are only interested in how you perceive your body in certain cases, and, therefore, only in sensations, feelings or impressions, and not in your knowledge on certain phenomena. For example, do not put items next to each other just because they relate to the same body part (e.g. *Dead Hand* and *Cold Hands*); you can put them next to each other only if you think that the sensations accompanying these experiences are similar. You can feel free to ask the researcher if something in the instructions is not clear. If you are ready, press the spacebar to start the experiment. "

For Review Only

Supplementary Table S3. Questionnaire items used for measurement of the subjective strength of the Rubber Hand Illusion and the Arm Immobilization Suggestion.

	**Rubber Hand Illusion**	**Arm Immobilization Suggestion**
	During the block I felt as if...	
Q1.	…the rubber hand belonged to me.	…my hand did not belong to me.
Q2.	…the rubber hand was my hand.	…my hand was not my hand.
Q3.	…the rubber hand was a part of my body.	…my hand was not a part of my body.

For Review Only

Supplementary Material S4 - Crib Sheet

ENGLISH

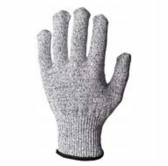

Empty glove. Simply an external object in the world – an object that is not part of you, in the same way as a desk, a lamp or a computer mouse are not. No sensations flow from the glove you have removed and you cannot move it directly. You feel and know that it is NOT your body.

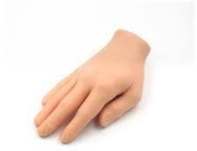

Rubber Hand (Exp. 1; Exp. 2 – experimental condition). The way the rubber hand was felt during the experiment. There are no right or wrong answers here, individual experiences can vary significantly!

Immobilized Arm (Exp. 2; control condition). The way your hand was felt during the experiment. There are no right or wrong answers here, individual experiences can vary significantly!

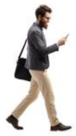

Normal body sensations. Touch is felt completely normally. Full ability to move and control your own body. The body can even seem 'transparent' as it operates without any problems. You feel and know that it is your body.

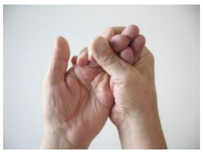

Cold hands. A feeling of very severe cold, usually felt in the lower or upper limbs. You may feel as if the blood supply to your limbs is restricted. The sensations from your hands are unnatural or significantly reduced, you may have trouble opening and closing your hands. There may be an overwhelming urge to "revive" your hands, for example by vigorously moving and massaging them.

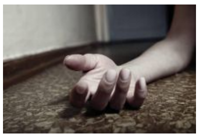

"Dead" hand. A completely or almost completely numb, inert hand. The experience most often occurs at night when the blood supply to the hand is cut off by a heavy, sleeping body. Temporary

For Review Only

loss or significant reduction in tactile sensations and ability to control the hand. Tingling or other unusual sensations may occur.

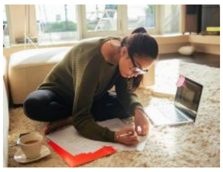

Sitting cross-legged. You have been sitting cross-legged for so long that you can no longer feel your legs. You may also feel a tingling or stiffness in your legs. There may be an overwhelming urge to move your legs to see if they are still there.

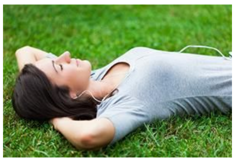

Deep relaxation. It can happen to you after a very good rest, or it can be the result of a conscious practice, such as body scanning or meditation. The body feels pleasantly heavy, soft and relaxed. You may feel energy and sensations that you do not pay attention to on a daily basis.

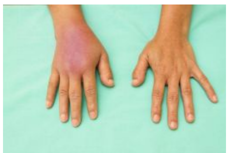

Swelling. Swelling on the part of your body resulting from a minor injury. The swollen part may be irritable and associated with unusual sensations. You may feel that something strange has grown on a part of your body.

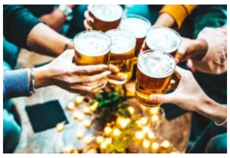

Alcohol intoxication. A moderate state of being under the influence of alcohol. The body 'listens to you' less than usual – you may be a little clumsy or bump on objects in the environment. At the same time, the body is less inhibited and moves quickly into action – for example, you may find it easier to dance. Bodily sensations may feel different, as slightly subdued or out of your field of attention.

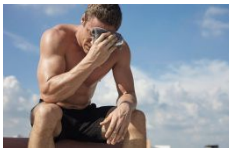

Post-workout. The body is warmed up and relaxed, but it is also very tired. You may feel that your body is not as dynamic as it was during the workout and 'does not want' to move.

For Review Only

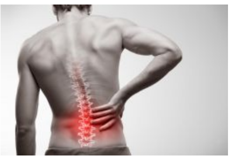

Pinched nerve. You may feel intense tingling or numbness associated with a compressed nerve. Sensations may feel like an overstimulation or strangely 'artificial'.

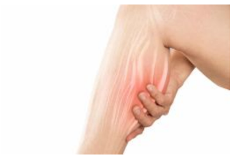

Muscle spasm. A strong muscle flexion, independent of your will, which can be painful or significantly restrict the mobility of a particular body part. You may need to massage the muscle and slowly 'check' whether it is able to relax.

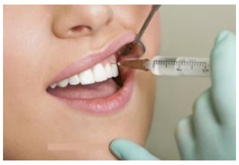

Local anesthesia. Weakening of sensations from a particular body part associated with an anesthetic drug injection (e.g. at the dentist). It entails a complete absence or significant weakening of tactile sensations and numbness. You may experience a recurring desire to touch the anesthetized area to check whether it is still in place.

POLISH (ORIGINAL)

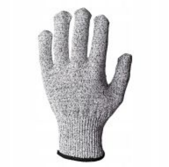

Pusta rękawiczka. Po prostu obiekt zewnętrzny w świecie – przedmiot nie będący częścią Ciebie, tak samo jak biurko, lampka czy mysz komputerowa. Ze zdjętej rękawiczki nie płyną żadne doznania i nie możesz nią bezpośrednio poruszać. Czujesz i wiesz, że to nie jest Twoje ciało.

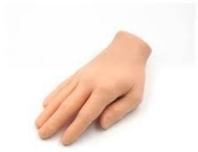

Rubber Hand. Sposób, w jaki była odczuwana gumowa ręka podczas eksperymentu. Nie ma tutaj dobrych lub złych odpowiedzi, wrażenia poszczególnych osób mogą się znacząco różnić!

Unieruchomiona ręka. Sposób, w jaki była odczuwana Twoja ręka podczas eksperymentu. Nie ma tutaj dobrych lub złych odpowiedzi, wrażenia poszczególnych osób mogą się znacząco różnić!

For Review Only

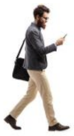

Normalne poczucie ciała. Dotyk odczuwany jest zupełnie normalnie. Pełna zdolność do poruszania się i kontrolowania własnego ciała. Ciało może wydawać się wręcz “przejrzyste”, ponieważ działa bez zakłóceń. Czujesz i wiesz, że to Twoje ciało.

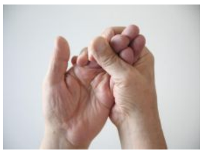

Zimne ręce. Uczucie bardzo silnego zimna, zazwyczaj odczuwane w kończynach dolnych lub górnych. Możesz czuć, jakby dopływ krwi do kończyn był ograniczony. Wrażenia z Twoich rąk są nienaturalne lub znacząco osłabione, możesz mieć problem z otwieraniem i zamykaniem dłoni. Może występować przemożna chęć “ożywienia” Twoich rąk, na przykład poprzez intensywne poruszanie i masowanie.

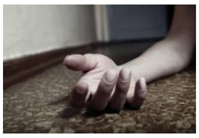

“Martwa” ręka. Zupełnie lub prawie zupełnie bezwładna ręka. Doświadczenie najczęściej wydarza się w nocy, kiedy dopływ krwi do ręki jest odcięty przez ciężkie, śpiące ciało. Chwilowy zanik lub znaczące ograniczenie doznań dotykowych i możliwości kontroli ręki. Może pojawić się mrowienie lub inne nietypowe doznania.

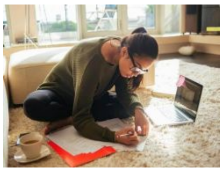

Siedzenie “po turecku”. Siedziałeś/aś po turecku tak długo, że aż przestałeś/aś odczuwać swoje nogi. Możesz również odczuwać mrowienie lub ścierpnięcie nóg. Może pojawić się przemożna chęć poruszania nogami w celu sprawdzenia czy nadal tam są.

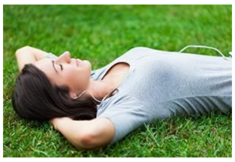

Głęboki relaks. Może przydarzyć Ci się podczas bardzo udanego odpoczynku lub być efektem świadomej praktyki – np. skanowania ciała lub medytacji. Ciało jest przyjemnie ciężkie, miękkie i

For Review Only

rozluźnione. Możesz czuć, że pojawiają się energia i doznania, na które na co dzień nie zwracasz uwagi.

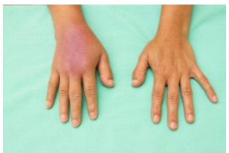

Opuchlizna. Będący efektem punktowego urazu obrzęk na Twojej części ciała. Opuchnięta część może być drażliwa i powiązana z nietypowymi doznaniami. Możesz czuć, że coś dziwnie obcego wyrosło na części Twojego ciała.

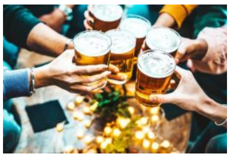

Upojenie alkoholowe. Umiarkowany stan bycia pod wpływem alkoholu. Ciało "mniej Cię słucha" niż zazwyczaj – możesz być trochę niezborny lub wpadać na przedmioty w otoczeniu. Jednocześnie ciało jest mniej zahamowane i szybko przechodzi do działania – np. może Ci się łatwiej tańczyć. Doznania cielesne mogą być odczuwane inaczej, jako nieco stłumione lub poza polem uwagi.

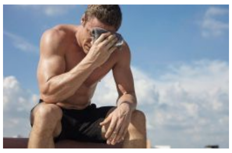

Po treningu. Ciało jest rozgrzane i zrelaksowane, ale jest także bardzo zmęczone. Możesz czuć, że Twoje ciało nie jest tak dynamiczne jak podczas treningu i "nie chce" się ruszać.

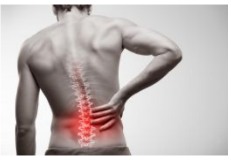

Uciśnięty nerw. Możesz czuć intensywne mrowienie lub drętwienie związane z uciśnięciem nerwu. Wrażenia mogą być odczuwane jako nadmiar stymulacji lub jako dziwnie "sztuczne".

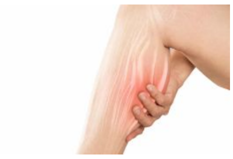

Skurcz mięśnia. Silne napięcie mięśnia, niezależne od Twojej woli, które może być bolesne lub znacząco ograniczać ruchomość danej części ciała. Może pojawić się potrzeba rozmasowania mięśnia i powolnego "sprawdzania" czy jest już w stanie się rozluźnić.

For Review Only

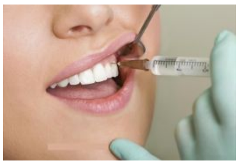

Znieczulenie miejscowe. Osłabienie wrażeń z danej części ciała związane z zastrzykiem zawierającym lek znieczulający (np. u dentysty). Towarzyszy mu całkowity brak lub znaczne osłabienie wrażeń dotykowych oraz odrętwienie. Możesz odczuwać powracającą chęć dotykania znieczulonego miejsca w celu sprawdzenia czy dalej jest na swoim miejscu.

For Review Only

Supplementary Material S5 - Familiarity Scale

1 – unfamiliar experience, has never happened to me

2 – experience happens to me occasionally, I can hardly imagine it

3 – experience happens to me now and then, I can imagine the gist of it

4 – experience happens to me quite often, I can imagine it relatively accurately

5 – the experience is very familiar to me, I can imagine it very accurately

For Review Only
